# Morphological, physiological, and transcriptional responses to low nitrogen stress in *Populus deltoides* Marsh. clones with contrasting nitrogen use efficiency

**DOI:** 10.1186/s12864-021-07991-7

**Published:** 2021-09-27

**Authors:** Cun Chen, Yanguang Chu, Qinjun Huang, Weixi Zhang, Changjun Ding, Jing Zhang, Bo Li, Tengqian Zhang, Zhenghong Li, Xiaohua Su

**Affiliations:** 1 State Key Laboratory of Tree Genetics and Breeding, Research Institute of Forestry, Chinese Academy of Forestry, Beijing, China; 2 Key Laboratory of Tree Breeding and Cultivation, State Forestry and Grassland Administration, Beijing, China; 3 Co-Innovation Center for Sustainable Forestry in Southern China, Nanjing Forestry University, Nanjing, Jiangsu Province China

**Keywords:** Nitrogen deficiency, Nitrogen use efficiency, Gene expression, *Populus deltoides* Marsh.

## Abstract

**Background:**

Nitrogen (N) is one of the main factors limiting the wood yield in poplar cultivation. Understanding the molecular mechanism of N utilization could play a guiding role in improving the nitrogen use efficiency (NUE) of poplar.

**Results:**

In this study, three N-efficient genotypes (A1-A3) and three N-inefficient genotypes (C1-C3) of *Populus deltoides* were cultured under low N stress (5 μM NH_4_NO_3_) and normal N supply (750 μM NH_4_NO_3_). The dry matter mass, leaf morphology, and chlorophyll content of both genotypes decreased under N starvation. The low nitrogen adaptation coefficients of the leaves and stems biomass of group A were significantly higher than those of group C (*p* < 0.05). Interestingly, N starvation induced fine root growth in group A, but not in group C. Next, a detailed time-course analysis of enzyme activities and gene expression in leaves identified 2062 specifically differentially expressed genes (DEGs) in group A and 1118 in group C. Moreover, the sensitivity to N starvation of group A was weak, and DEGs related to hormone signal transduction and stimulus response played an important role in the low N response this group. Weighted gene co-expression network analysis identified genes related to membranes, catalytic activity, enzymatic activity, and response to stresses that might be critical for poplar’s adaption to N starvation and these genes participated in the negative regulation of various biological processes. Finally, ten influential hub genes and twelve transcription factors were identified in the response to N starvation. Among them, four hub genes were related to programmed cell death and the defense response, and *PodelWRKY18*, with high connectivity, was involved in plant signal transduction. The expression of hub genes increased gradually with the extension of low N stress time, and the expression changes in group A were more obvious than those in group C.

**Conclusions:**

Under N starvation, group A showed stronger adaptability and better NUE than group C in terms of morphology and physiology. The discovery of hub genes and transcription factors might provide new information for the analysis of the molecular mechanism of NUE and its improvement in poplar.

**Supplementary Information:**

The online version contains supplementary material available at 10.1186/s12864-021-07991-7.

## Background

Nitrogen (N) is an essential macronutrient for plant growth and development, and is an important constituent of nucleic acids, proteins, hormones, and chlorophyll. Nitrogen also participates in a variety of biological processes as a signal to regulate the growth of aboveground and underground parts of plants [1–3]. Meanwhile, N is the main limiting factor of plant productivity and crop yield [4]. Therefore, the application of N fertilizer in agricultural production has become the main method to improve crop yield; however, in practice, only about 30 to 40% of the applied N fertilizer is absorbed by crops and used effectively; the rest is retained in the soil or integrated into water resources, which not only wastes resources but also affects the nutrient balance, resulting in environmental pollution [1, 5–7]. Moreover, it is not feasible to increase the timber yield of perennial trees by fertilization; therefore, it is particularly important to improve the nitrogen use efficiency (NUE) of plants for crops, especially for trees.

NUE is a comprehensive characteristic of the interaction between the available N content in the plant growth environment and various biological processes, including absorption, transport, assimilation, signal transduction, and regulation [8]. In general, NUE mainly includes two aspects: N uptake efficiency (NUpE) and N utilization efficiency (NUtE). N-efficient plants should have a high NUpE and a high NUtE [9]. The absorption of N is the first step in the utilization of N, and the ability of NUpE directly determines the NUE of plants [10, 11]. Plants usually acquire N from the soil in the form of NO_3_^−^ and NH_4_^+^ by the roots, with the help of specific transporters, including nitrate transporters (NRTs) and ammonium transporters (AMTs) [12]. Poplar roots could acquire phenylalanine as a sole N source to support plant growth [13]. In contrast to herbaceous plants, N nutrition of poplar is maintained by seasonal and internal N circulation, and the vegetative storage proteins (BSP, WIN4, and PNI288) play a role in N storage and seasonal N cycling in poplar [14]. Recent studies on NUE mainly focused on the root system, and different zones of poplar roots showed distinct capacities for N absorption and assimilation because of differentially expressed microRNA (miRNA)-target pairs [15]. The results of transcriptome and metabolome analysis of poplar roots in response to N deficiency showed that males had a better osmotic adjustment ability and higher NUE, indicating a better stress tolerance ability compared with that of females [16, 17]. We believe that there is a close relationship between roots and leaves in the form of a ‘source-sink’. In addition, gene expression and physiological activity in the leaves, especially photosynthesis, play an important role in NUtE [18]. In previous studies, key genes involved in the response to low N stress in leaves were ignored.

Studying the mechanism of NUE and selecting N-efficient genotypes, are effective strategies to achieve a stabilized yield and high NUE. However, because of the promotion of single cultivation in crop and forest production, the genetic diversity of NUE has become narrower, representing a bottleneck for the genetic improvement of NUE [19]. The most plausible approach is to excavate favorable natural genetic variation from existing germplasm resources under low N stress, to study the molecular genetic basis of favorable variation, and make full use of natural variation, thus laying the foundation for the selection of N-efficient genotypes and the improvement of NUE-related traits [8, 20].

In previous studies, we found that high-throughput transcriptome sequencing (RNA-seq) technology could effectively identify differentially expressed genes (DEGs) under low N stress, and was useful to mine key regulatory genes closely related to NUE [21–23]. In some crops, two genotypes with different NUEs were selected to explore the key genes involved in the regulation of N efficient utilization [9, 24, 25]. The same strategy had been used in the study of N metabolism of poplar [26]. Luo et al. analyzed the differences between two contrasting poplar species [a fast-growing species (*P. alba* × *P. glandulosa*, Pg) and a slow-growing species (*P. popularis*, Pp)] at the transcriptional level under low N stress, and found that 18 genes involved in N metabolism showed stronger responses to transcriptional regulation in the roots and leaves of Pp than in those of Pg [27]. However, there have been no studies on N metabolism of *Populus deltoides* Marsh. (*P. deltoides*) using two completely different NUEs. The construction and analysis of gene-to-gene regulatory networks are useful to discover the potential key regulators among DEGs, and this method has been applied in many studies to identify key regulatory genes in a network [28–30]. Liu et al. found that circular RNAs play an essential role in modulating wood anatomical and chemical properties of poplar to adapt to low N environment through circRNAs-miRNAs-mRNAs networks [31]. Wei et al. analyzed the temporal patterns of the DEGs identified in poplar (*P. tremula* × *P. alba*) roots to low N stress, and identified presence of conserved signaling mechanisms triggered by low N stress. Moreover, a sub-network centered on the transcription factor *PtaNAC1* was revealed, indicating that there were some hierarchically structured networks centered on key genes in roots in response to low N availability [32]. However, there has been little analysis on the timeliness of the response to low N stress of genes in the leaves of *P. deltoides*.

*Populus* species are fast-growing and easily propagated woody plants, which play an important role in ecological protection and wood production. Poplar is one of the main afforestation tree species in the middle latitude of the world. However, with the gradual expansion of the poplar plantation area, they often grow in poor lands where soil N is limited [33, 34]. Therefore, it is necessary to explore the mechanism of poplar’s N efficient utilization and improve its NUE. *P. deltoides*, with high genetic diversity, is often used in research into poplar hybrid breeding as a parent [35]. In our previous study, 338 genotypes of *P. deltoides* were classified according to their NUE, and 26 N-efficient genotypes and 24 N-inefficient genotypes were obtained [36]. In the present study, three genotypes were selected from the two contrasting groups, respectively, and were treated with low N stress for 40 days. The morphological and physiological differences between the genotypes were analyzed, and a detailed time-course analysis of enzyme activities and gene expression related to N metabolism in leaves was carried out to analyze the difference of two contrast genotypes in response to low N stress. The key genes or transcription factors (TFs) responding to low N stress were mined using weighted gene co-expression network analysis (WGCNA). The results of this study would provide a valuable resource to further develop strategies to improve the NUE in poplar.

## Results

### Differences in growth characteristics under low N stress

**Fig. 1:**
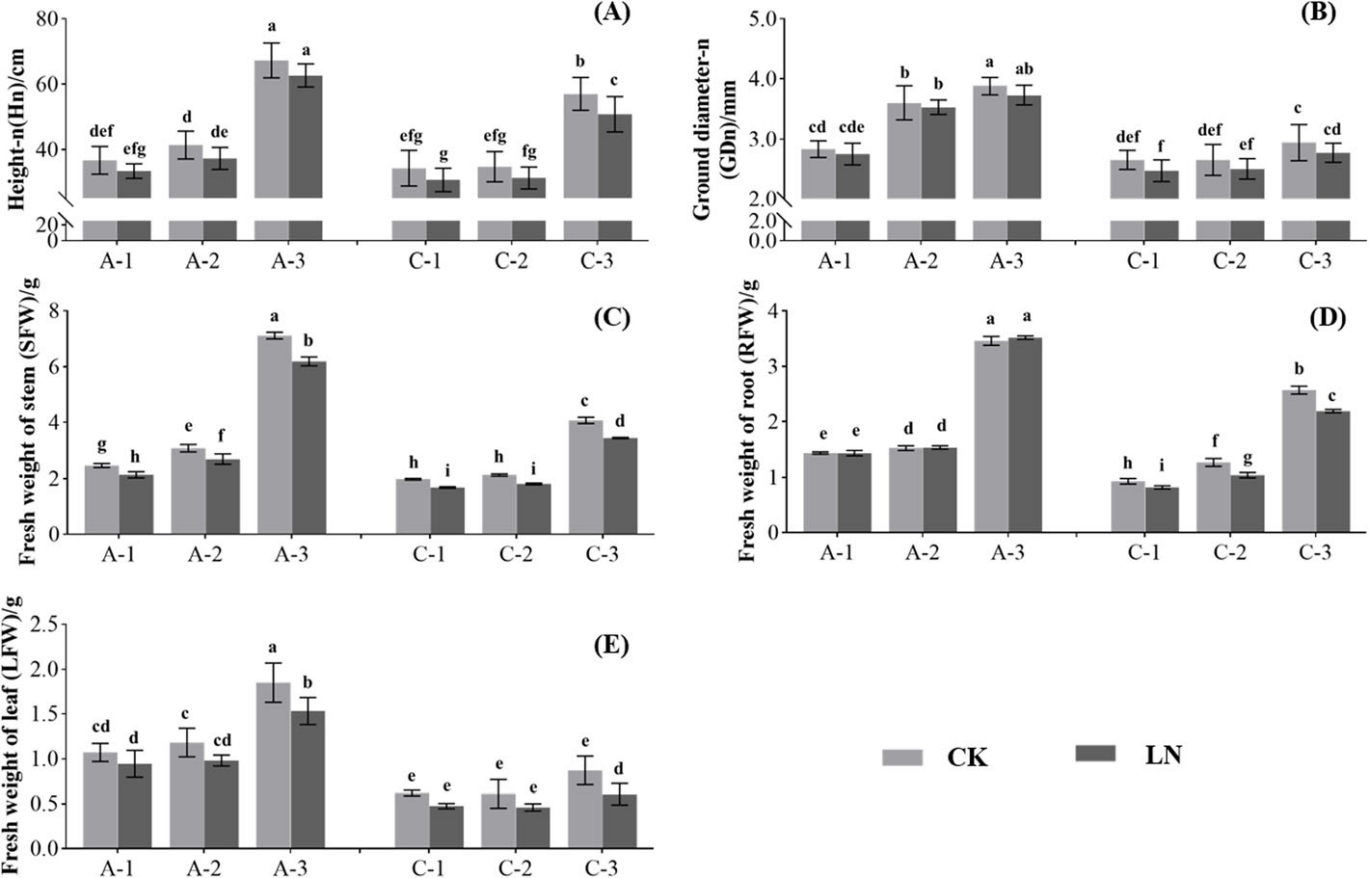
Effects of low N stress on growth traits of N-efficient (A-1, A-2, and A-3) and N-inefficient (C-1, C-2, and C-3) genotypes. Different letters above the column indicate significant differences between groups (*p* < 0.05). (A) Height after treatment (Height-n, Hn); (B) Ground diameter after treatment (Ground diameter-n, GDn); (C) Fresh weight of the stem (SFW); (D) Fresh weight of the root (RFW); (E) Fresh weight of the leaf (LFW)

Under the normal N supply (CK) treatment, the plant height of the three N-efficient genotypes were 36.73, 41.43, and 67.20 cm, respectively, while under low nitrogen (LN) treatment, the plant heights of the three genotypes were 33.44, 37.32, and 62.62 cm, respectively. Compared with CK treatment, the average plant height decreased by 8.56% under LN treatment after 40 days. For the N-inefficient genotypes, the average plant height decreased by 10.39%(Fig. 1A). The average ground diameter (GDn) of N-efficient genotypes decreased by 2.90%, while the GDn of the N-inefficient genotypes decreased by 5.94% (Fig. 1B). Similarly, under LN treatment, the average fresh weight of the stem (SFW) and dry weight of the stem (SDW) of N-efficient plants decreased by 12.89 and 15.74%, respectively, while the SFW and SDW of the N-inefficient plants decreased by 15.22 and 23.89%, respectively (Fig. 1C). Notably, the fresh weight of the roots (RFW) of the three N-efficient genotypes were 1.435, 1.525, and 3.455 g under CK treatment, respectively, while in the LN treatment, the RFW of the three genotypes increased slightly, to 1.440, 1.535, and 3.515 g, respectively. While the RFW of the N-inefficient genotypes decreased significantly (*p* < 0.05). By contrast, the dry weight of the roots (RDW) of the N-efficient and N-inefficient genotypes (except the C-1 clone) was reduced significantly under LN treatment (*p* < 0.05, Fig. 1D, Fig. S1 and S2). Moreover, the leaf fresh weight (LFW) and leaf dry weight (LDW) of a single leaf of the N-efficient and N-inefficient genotypes decreased under LN treatment, and the values of LFW and LDW of the N-efficient genotypes were higher than those of the N-inefficient genotypes under CK or LN treatment (*p* < 0.05, Fig. 1E, Fig. S1 and S2). As shown in Table S1, the low N adaptation coefficient (LNAC) of GDN, SFW, SDW, RFW, LFW, and LDW of the N-efficient genotypes was significantly higher than that of the N-inefficient genotypes (*p* < 0.05).

### Differences in leaf morphology and chlorophyll content under low N stress

**Fig. 2:**
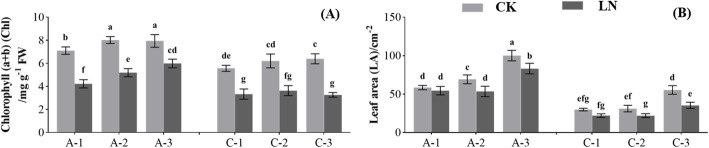
Effects of low N stress on leaf morphology and chlorophyll content of N-efficient (A-1, A-2, and A-3) and N-inefficient (C-1, C-2, and C-3) genotypes. Different letters above the columns indicate significant differences between the groups (*p* < 0.05). (A) Chlorophyll (a + b) (Chl); (B) Leaf area (LA)

The contents of chlorophyll a (Chl a), chlorophyll b (Chl b), carotenoids (Car), and total chlorophyll (Chl) of plants (N-efficient and N-inefficient genotypes) under LN treatment were significantly lower than those under CK treatment (*p* < 0.05, Fig. 2A and Fig. S1). Besides, the chlorophyll contents (Chl a, Chl b, Car, and Chl) of the N-efficient genotypes were higher than those of the N-inefficient genotypes under LN or CK treatment (*p* < 0.05, Fig. 2A and Fig. S1). For example, under LN treatment, the Chl of three N-efficient genotypes were 4.22, 5.19, and 5.99 mg·g^− 1^, respectively, while the Chl of three N-inefficient genotypes were 3.26, 3.33, and 3.63 mg·g^− 1^, respectively (Fig. 2A). Compared with CK treatment, leaf morphological traits [leaf length (LL), leaf width (LW), and leaf area (LA)] were reduced under LN treatment (Fig. 2B, Fig. S1 and S2). The LL, LW, and LA of the N-efficient genotypes decreased by 9.43, 8.99, and 15.50%, respectively, while those of the N-inefficient genotypes decreased by 15.22, 14.64, and 30.58%, respectively. In particular, under LN treatment, the LA of the three N-efficient genotypes were 53.42, 54.49, and 83.10 cm^2^, which were higher than those of the three N-inefficient genotypes (*p* < 0.05, Fig. 2B). The average LNAC values of Chl a, Chl b, Car, Chl, LL, LW, and LA of the N-efficient genotypes were higher than those of the N-inefficient genotypes,; however, the differences were not significant (Table S1).

### Differences in the response of enzyme activities under low N stress

To study the differences between the two contrasting genotypes in response to low N stress, we measured the enzyme activities (nitrate reductase (NR), glutamine synthetase (GS), glutamate dehydrogenase (GDH), and glutamine oxoglutarate aminotransferase (GOGAT)), total amino acid contents (AAs), and soluble sugar contents (SSs) of mixed leaf samples at seven time points (T0, T1, T2, T3, T4, T5, and T6 represent 0, 3, 5, 10, 20, 30, and 40 days of N treatment, respectively). As shown in Fig. S3, the enzyme activities in leaves of the N-efficient (A) and N-inefficient (C) genotypes were inhibited under LN treatment and the AA content decreased significantly after 5 days (T2) of LN treatment (*p* < 0.05). At T0, T2, T3, T5, and T6 of LN treatment, the NR activity of genotype A was higher than that of genotype C (*p* < 0.05), and the NR activity reached the lowest at T4 (Fig. S3, Table S2). With increasing treatment time, the activities of GS and GOGAT of genotypes A and C increased first and then decreased, with the turning point of change mostly occurring at T2 (except for the change of GS activity of genotype A). At T5 and T6, the activities of GS and GOGAT of genotypes A and C were decreased significantly under LN treatment (*p* < 0.05, Fig. S3, Table S2). The activity of GS in genotype A was higher than that in genotype C before LN treatment (*p* < 0.05). However, after 5 days (T2), there was no significant difference in GS activities between genotypes A and C, and the GS activity of genotype A was significantly lower than that of genotype C after 40 days (T6) of LN treatment (*p* < 0.05, Table S2). Besides, the GDH activity of genotype A decreased first and then increased, while that of genotype C decreased continuously (Fig. S3). Under CK treatment, there was no significant difference in GDH activity between genotypes A and C (except at T0). After 20 days (T4) of LN treatment, the GDH activity of genotype A was significantly higher than that of genotype C (*p* < 0.05, Table S2). The AA content of genotypes A and C decreased continuously in response to low N stress (Fig. S3). The AA content of genotypes A and C showed no significant difference at the same time points under CK treatment (except T2); however, the AA content of genotype A was higher than that of genotype C at T3, T4, and T6 under LN treatment (*p* < 0.05, Table S2). In particular, at the beginning of low N stress, the SS content of genotype A decreased and then increased gradually, while genotype C showed the opposite response (Fig. S3).

### Evaluation of RNA sequencing data

According to the change trends of enzyme activities, AAs, and SSs in the process of LN treatment, the mixed leaf samples of N-efficient (A) and N-inefficient (C) genotypes at 0 (T0), 5 (T2), 20 (T4), and 40 (T6) days after LN treatment, with three biological replicates (T0-A-1, T0-A-2, T0-A-3, T0-C-1, T0-C-2, T0-C-3, T2-LN-A-1, T2-LN-A-2, T2-LN-A-3, T2-LN-C-1, T2-LN-C-2, T2-LN-C-3, T4-LN-A-1, T4-LN-A-2, T4-LN-A-3, T4-LN-C-1, T4-LN-C-2, T4-LN-C-3, T6-LN-A-1, T6-LN-A-2, T6-LN-A-3, T6-LN-C-1, T6-LN-C-2, and T6-LN-C-3) were selected for transcriptome sequencing analysis. A total of 36,866,796–61,136,254 clean reads were generated, of which approximately 76.89–87.63% were uniquely mapped to the genome of *P. deltoides* (Table S3). The Pearson correlation coefficient between the biological replicates ranged from 0.9810 to 0.9977 (Fig. S4), implying that the RNA-seq data were highly reliable.

### DEGs between N-efficient and N-inefficient genotypes under low N stress

To study the differences of response to low N stress of the two genotypes (A and C), we detected the DEGs between A and C comparison groups at four-time points (T0-C vs. T0-A, T2-LN-C vs. T2-LN-A, T4-LN-C vs. T4-LN-A, and T6-LN-C vs. T6-LN-A), and 2383; 1243; 1292; and 3055 DEGs were detected, respectively. Compared with genotype C, 1149 DEGs were upregulated and 1906 genes were downregulated in genotype A at T6 (Fig. S5). As shown in the Venn diagram (Fig. S5), there were 397 common DEGs at T0, T2, T4, and T6 between genotypes A and C, and 972, 225, 224, and 1578 specific DEGs at T0, T2, T4, and T6, respectively.

**Fig. 3:**
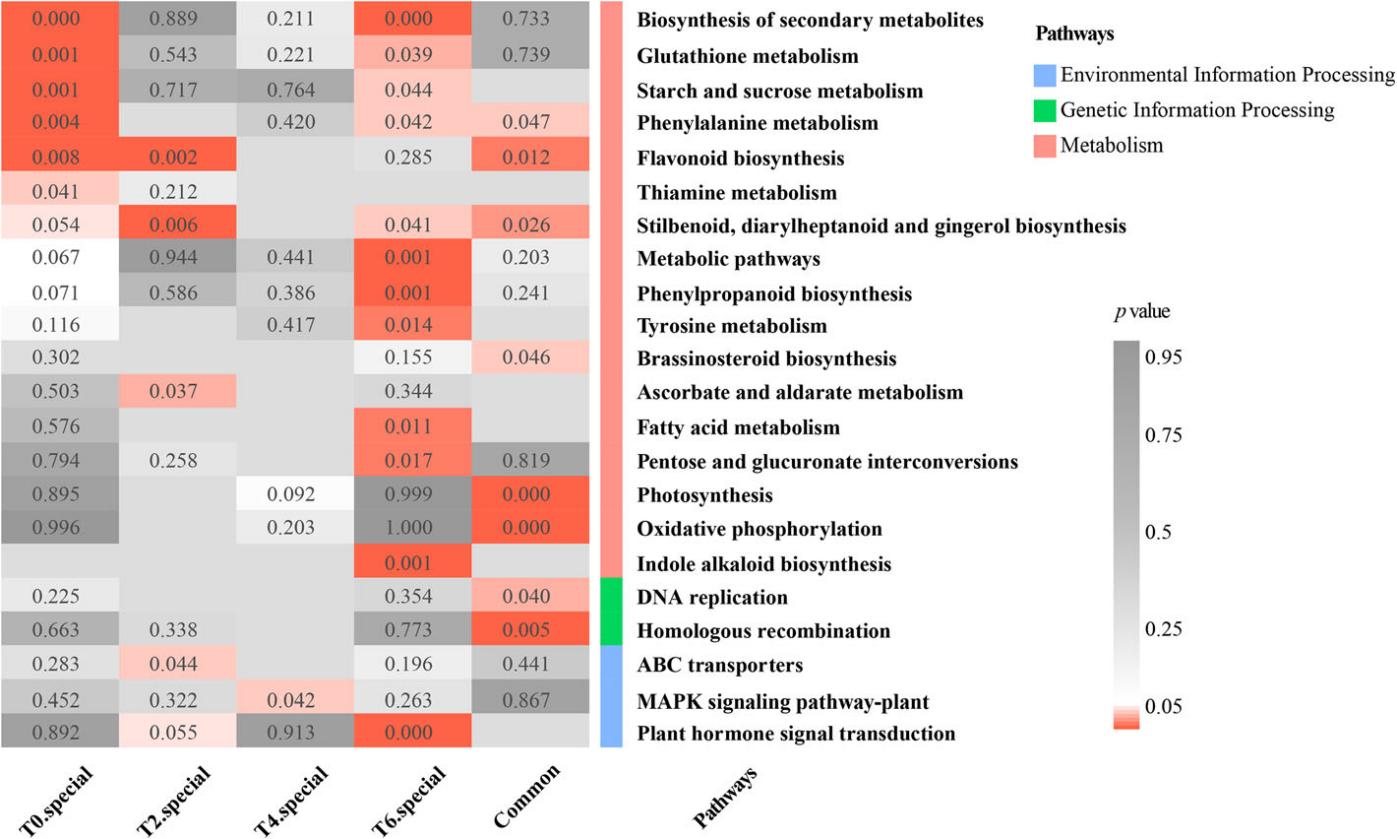
Results of the Kyoto Encyclopedia of Genes and Genomes (KEGG) pathway enrichment analysis of the special differentially expressed genes (DEGs) in genotype A at different time points and common DEGs in genotypes A and C during the response to low N stress. T0 special, T2 special, T4 special, and T6 special represent the specific DEGs at T0, T2, T4, and T6 in genotype A, respectively. “Common” indicates the common DEGs at T0, T2, T4, and T6 in genotypes A and C

At T0, T2, T4, and T6, the specific DEGs in genotype A were enriched for 40, 32, 34, and 42 Gene Ontology (GO) terms, and more DEGs were mainly enriched in the metabolic process, catalytic activity, and cell. Some DEGs were related to stimulus response in genotype A at different time points (Fig. S6). The Kyoto Encyclopedia of Genes and Genomes (KEGG) pathway enrichment analysis of specific and common genes at different time points between genotypes A and C showed that the specific DEGs at T0 were mainly enriched in the biosynthesis of secondary metabolites, glutathione metabolism, starch and sucrose metabolism, phenylalanine metabolism, flavonoid biosynthesis, and thiamine metabolism (Fig. 3). The 225 specific DEGs at T2 were specifically enriched for ascorbate and aldarate metabolism, and one environmental information processing category (ABC transporters). The distinct DEGs at T6 were specifically enriched for metabolism (metabolic pathways, phenylpropanoid biosynthesis, tyrosine metabolism, fatty acid metabolism, pentose and glucuronate interconversions, and indole alkaloid biosynthesis) and plant hormone signal transduction. The common DEGs were mainly enriched in photosynthesis, oxidative phosphorylation, DNA replication, and homologous recombination pathways. These results suggested that genotypes A and C have different response mechanisms to low N stress.

**Fig. 4:**
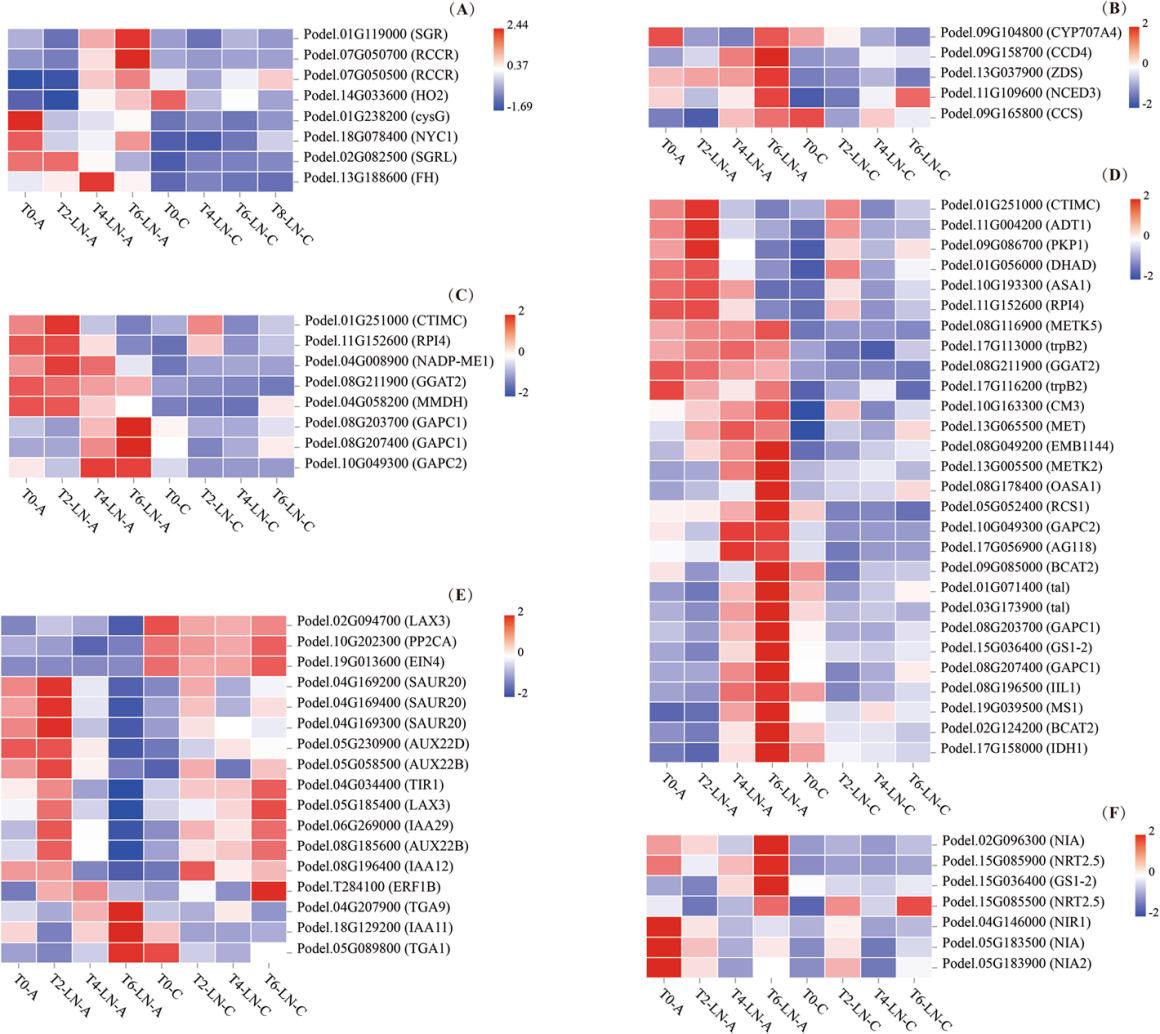
Expression analysis of differentially expressed genes (DEGs) related to porphyrin and chlorophyll metabolism (A), carotenoid biosynthesis (B), carbon fixation in photosynthetic organisms (C), biosynthesis of amino acids (D), plant hormone signal transduction (E), and nitrogen metabolism (F) in genotypes A and C during the response to low N stress

Eight DEGs related to porphyrin and chlorophyll metabolism, and five DEGs related to carotenoid biosynthesis were detected between genotypes A and C, their expression levels in genotype A were higher than those in genotype C after 20 days (T4) of LN treatment (Fig. 4A, B). During the LN treatment, the expression of eight DEGs involved in carbon fixation in photosynthetic organisms in genotype A were higher than those in genotype C (Fig. 4C). After 20 days of low N stress, 22 of 28 DEGs related to biosynthesis of amino acids were expressed at higher levels in genotype A than in genotype C (Fig. 4D). The expression patterns of genes related to plant hormone signal transduction were different between genotype A and genotype C in response to low N stress: 11 of 17 DEGs were expressed at lower levels in genotype A than in genotype C at 40 days (T6) of low N stress (Fig. 4E). Among the seven DEGs related to nitrogen metabolism, four genes were upregulated and three genes were downregulated in genotype A; however, their expression levels were higher than those in genotype C (Fig. 4F).

### DEGs during low N stress of N-efficient and N-inefficient genotypes

The DEGs of genotypes A and C at different time points under low N stress were determined to study the differences of response mechanisms of the two genotypes. Three different comparison groups of genotype A at the early stage (T0-A vs. T2-LN-A), the middle stage (T2-LN-A vs. T4-LN-A), and the late stage (T4-LN-A vs. T6-LN-A) were constructed, and 445 (272 upregulated and 173 downregulated), 2337 (1624 upregulated and 713 downregulated), and 692 (219 upregulated and 473 downregulated) DEGs were detected, respectively (Fig. S7). Three corresponding comparison groups of genotype C (early stage: T0-C vs. T2-LN-C, middle stage: T2-LN-C vs. T4-LN-C. and late-stage: T4-LN-C vs. T6-LN-C) were constructed, and 1635 (1231 upregulated and 404 downregulated), 598 (153 upregulated and 445 downregulated) and 226 (132 upregulated and 94 downregulated) DEGs were identified, respectively (Fig. S7). The results showed that the gene response of genotype A was mainly in the middle stage of low N stress, while that of genotype C was mainly in the early stage. There were 191, 319, and 50 common DEGs between genotypes A and C in the early, middle, and late stages of LN treatment, respectively. Notably, there were 926 common DEGs between genotypes A and C in response to low N stress; however, there were more specific DEGs in genotype A (2,062) than in genotype C (1,118; Fig. S7).

**Fig. 5:**
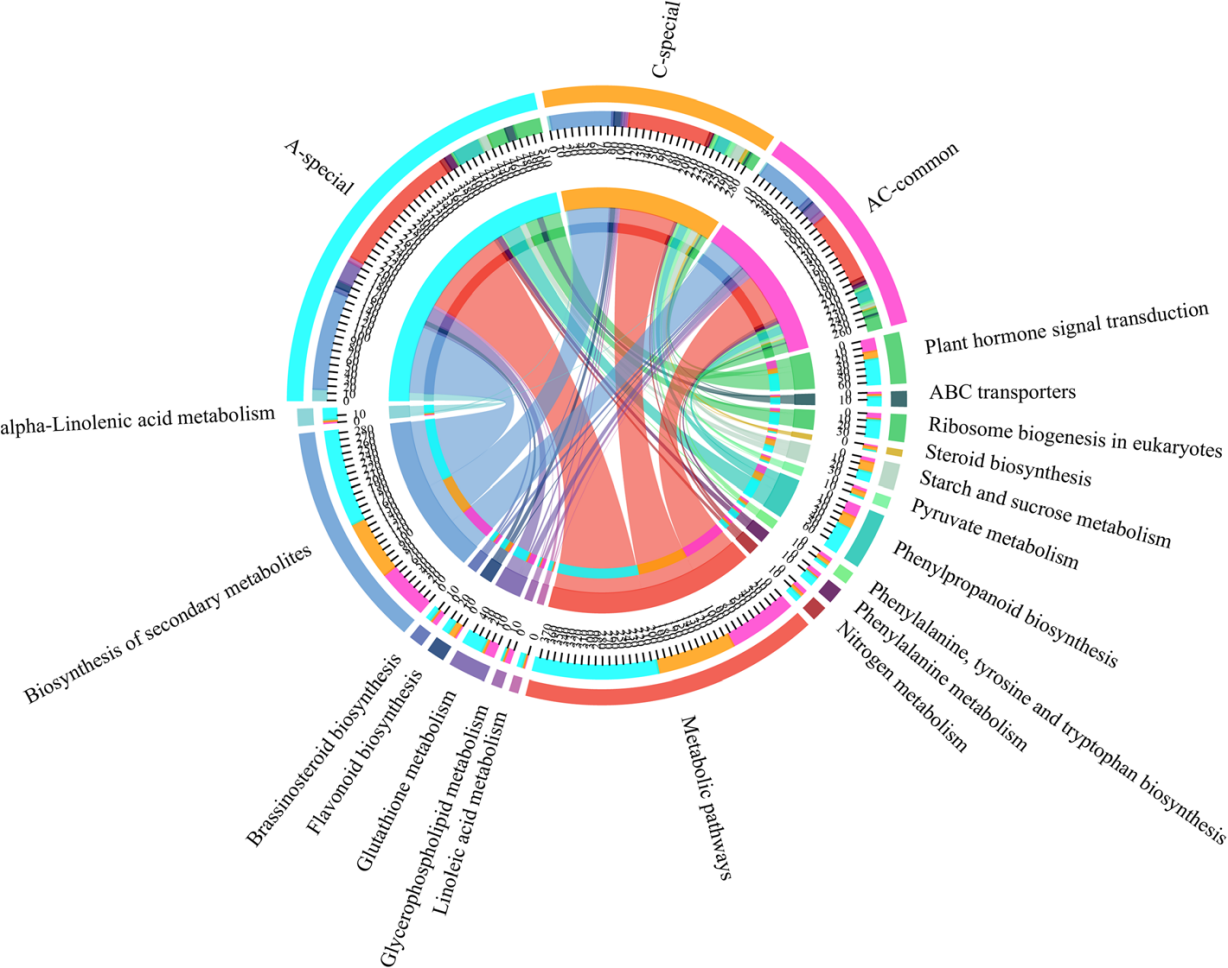
Results of the Kyoto Encyclopedia of Genes and Genomes (KEGG) pathway enrichment analysis of the special and common differentially expressed genes (DEGs) between genotypes A and C during the response to low N stress. A-special and C-special represent the specific DEGs in genotypes A and C, respectively. AC-common indicates the common DEGs in genotypes A and C

The specific DEGs in genotypes A were associated with protein kinase activity, protein phosphorylation, negative regulation of development process, and catalytic activity. By contrast, the DEGs specifically expressed in genotype C were enriched in microtubule-based process, intrinsic component of membrane, and membrane part (Fig. S8). Moreover, during the response of genotypes A and C to low N stress, the expression levels of genes related to metabolic pathways and biosynthesis of secondary metabolites changed significantly. The difference was that in genotype A, many DEGs were enriched in glutathione metabolism, phenylalanine metabolism, phenylpropanoid biosynthesis, ABC transporters, ribosome biogenesis in eukaryotes, and plant hormone signal transduction, whereas in genotype C, many DEGs were enriched in flavonoid biosynthesis, pyruvate metabolism, starch and sucrose metabolism, and steroid biosynthesis (Fig. 5).

### Trend analysis of DEGs

For genotypes A and C, DEGs that were detected in response to low N stress at four-time points were clustered into 20 profiles, among which 2988 DEGs of genotype A were significantly enriched in six profiles (profile 0, 6, 9, 10, 12, and 13), among which profile 12 contained the largest number of genes (1168), which was similar to the previous research results, i.e., the genes in genotype A mainly responded in the middle stage of low N stress, and less in the early and late stages (Fig. S9). For genotype C, 2044 DEGs were significantly enriched in four profiles (profile 2, 14, 15, and 17), among which profile 17 contained the largest number of genes (629), which showed that genes mainly responded at the early stage of treatment, and fewer genes participated in the middle and late stages (Fig. S9).

**Fig. 6:**
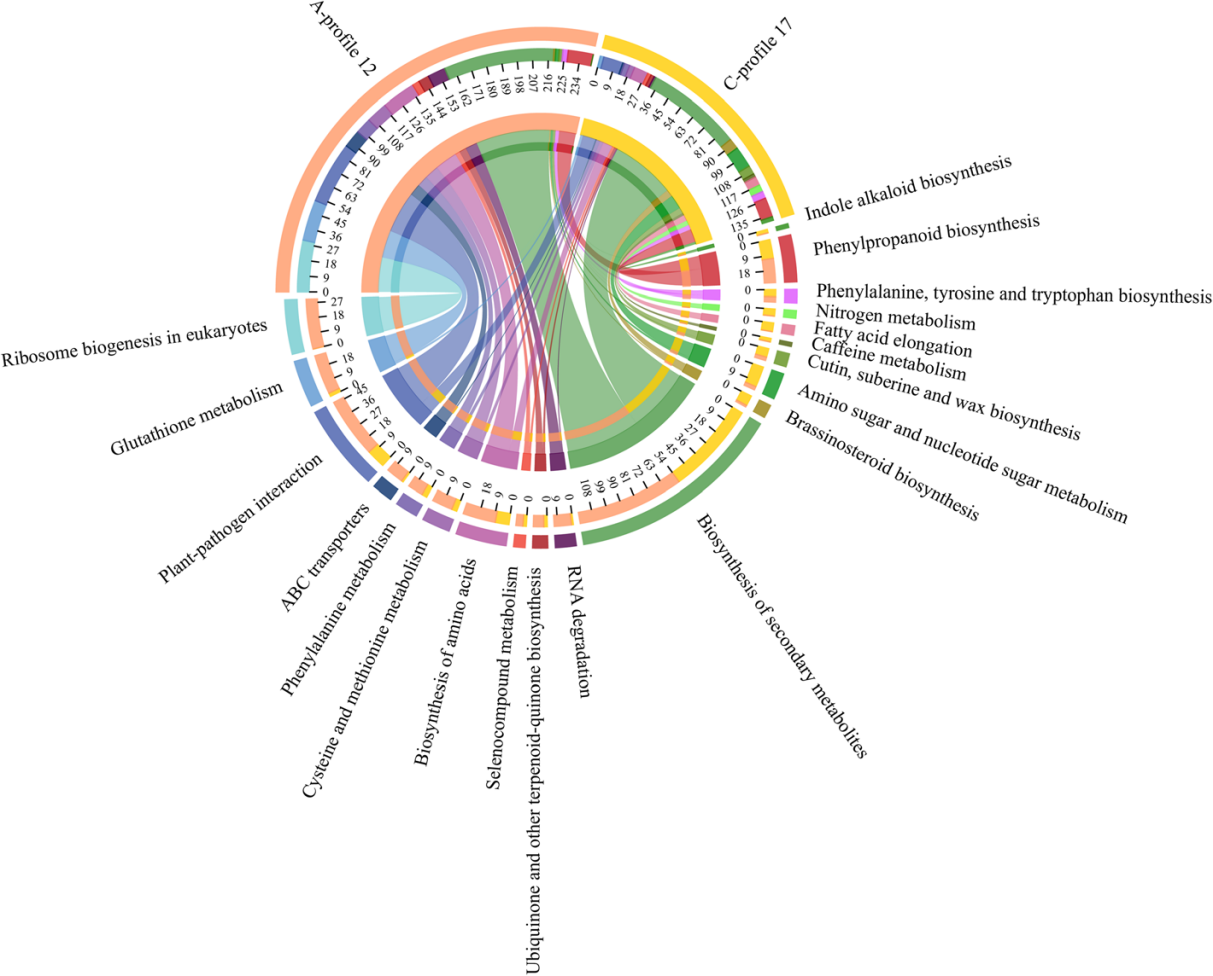
Results of the Kyoto Encyclopedia of Genes and Genomes (KEGG) pathway enrichment analysis of differentially expressed genes (DEGs) in profile 12 of genotype A (A-profile 12) and profile 17 of genotype C (C-profile 17). The 10 pathways on the left are the top 10 KEGG pathways with significantly enrichment in A-profile 12, and the 10 pathways on the right are the top 10 KEGG pathways with significantly enrichment in C-profile 17 (*p* < 0.05)

Genes in profile 12 of genotype A (A-profile 12) were mainly involved in kinase activity, transport, localization and binding of various substances, as well as the defense response, whereas the genes in profile 17 of genotype C (C-profile 17) were mainly related to membrane, microtubule, cytoskeletal part, and cytokinesis (Fig. S10). Many genes in A-profile 12 and C-profile 17 were enriched in the pathway of biosynthesis of secondary metabolites. Genes in A-profile 12 were related to the biological processes of N absorption, transformation, transport, and assimilation (glutathione metabolism, phenylalanine metabolism, cysteine, and methionine metabolism, biosynthesis of amino acids, and ABC transporters), while the genes in C-profile 17 were significantly enriched in brassinosteroid biosynthesis, amino sugar, and nucleotide sugar metabolism, fatty acid elongation, and N metabolism (Fig. 6).

### Identification of WGCNA modules associated with special traits

**Fig. 7:**
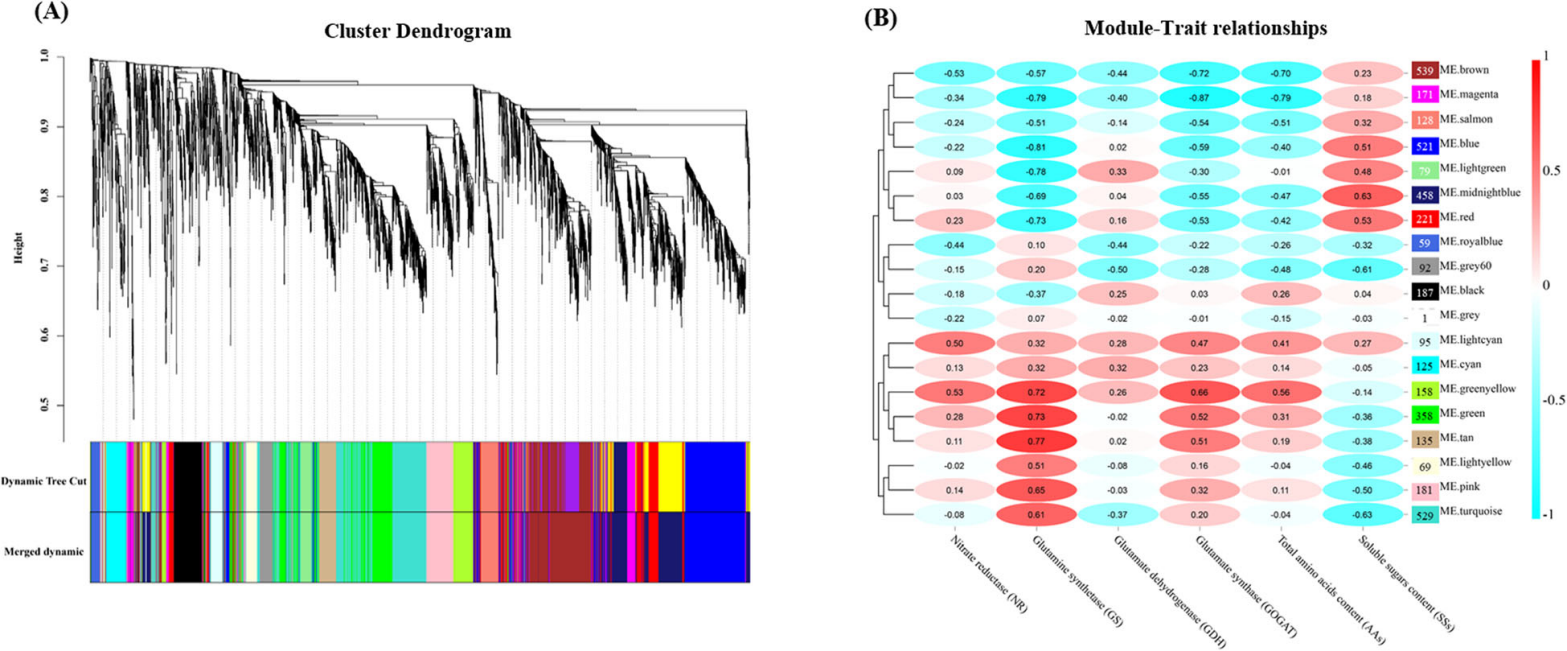
Weighted gene co-expression network analysis (WGCNA) of differentially expressed genes (DEGs) identified in genotypes A and C over three-time stages under low N stress. (A) Gene cluster dendrogram and 19 gene module divisions of DEGs, in which a major tree branch represents a module, and different colors represent different modules. (B) Correlation heatmap between modules and traits. Each column presents the experimental traits. The number in the oval box represents the correlation coefficient, which ranges from − 1 (cyan) to 1 (red). We set an absolute value of the correlation coefficient greater than 0.75 to indicate that there is a strong correlation between gene modules and traits. The number in the rectangular box on the right indicates the number of the genes contained in the corresponding gene module

A total of 24 leaf samples of genotypes A and C at four-time points (three biological repeats) during low N treatment were used to carry out WGCNA. Based on the results (Fig. 7A), 4106 genes were divided into 19 modules. Except for the grey module with only one gene, the other module sizes ranged from 59 (‘royal blue’) to 539 (‘brown’). Through correlation analysis between modules and experimental traits (NR, GS, GDH, GOGAT, AAs and SSs; Fig. 7B), it was found that the correlation coefficients between GS and ‘magenta’, ‘blue’, ‘light green’, and ‘tan’ were − 0.79, − 0.81, − 0.78, and 0.77, respectively. Moreover, the ‘magenta’ module was not only significantly related to GS, but also correlated highly with GOGAT and AAs (|r| > 0.75, *p* < 0.05). Therefore, we speculated that genes in the ‘magenta’ module might play a key role in the plant response to low N stress.

### Gene expression trends and function analysis of a specific module

**Fig. 8:**
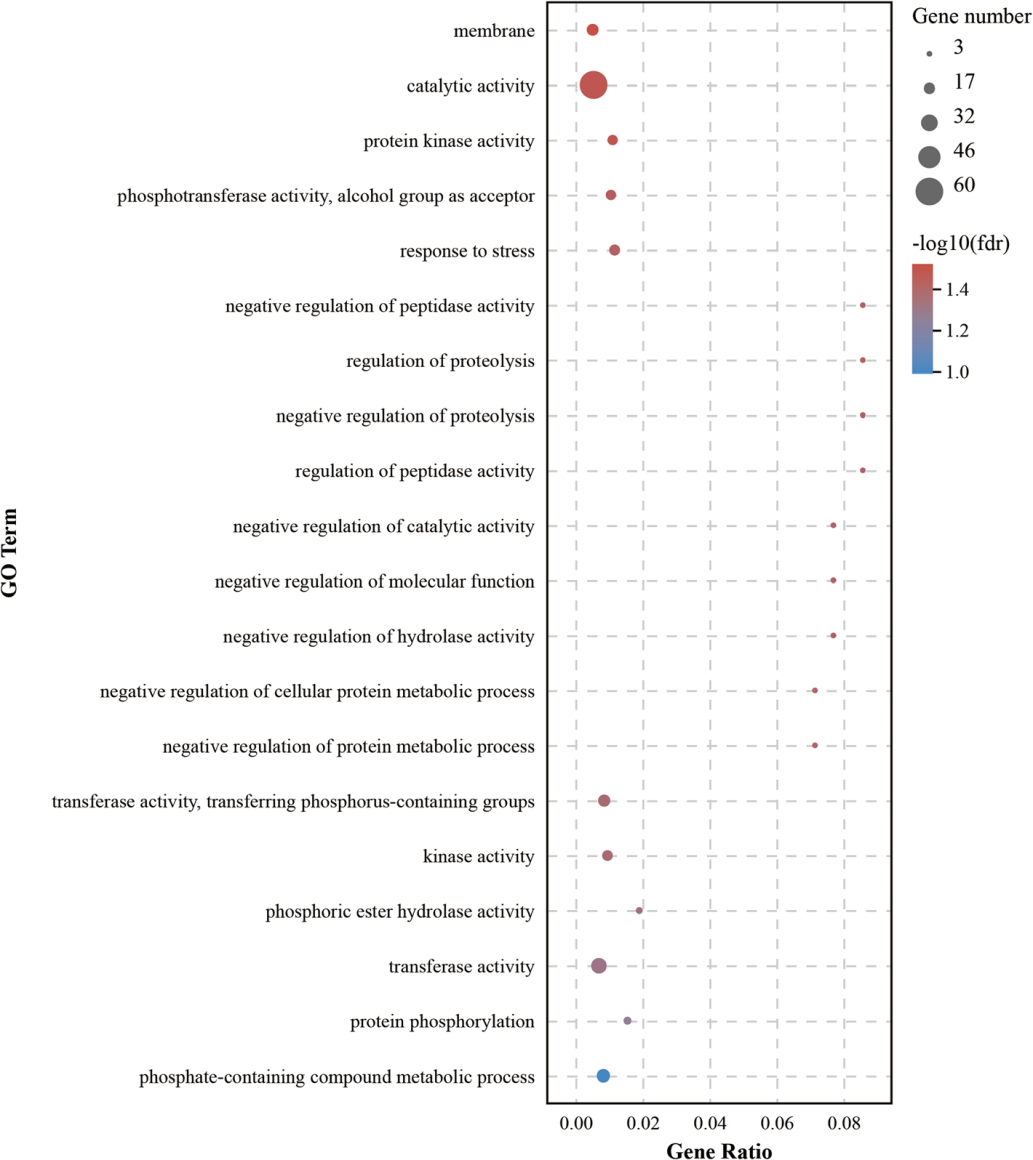
Top 20 gene ontology (GO) terms of genes in the ‘magenta’ module

The expression trends of genes in the ‘magenta’ module were similar in genotypes A and C, and most of the genes had low expression at T0, after which expression increased gradually with increasing treatment time of low N stress; however, the expression trends of a few other genes were the opposite. Moreover, the expression of these genes in genotype A was more obvious than that in genotype C (Fig. S11). An analysis of functional annotations (Gene Ontology terms) revealed that genes in the ‘magenta’ module are related to the membrane, catalytic activity, enzymatic activity, response to stress, and negative regulation of various biological processes (Fig. 8).

### Co-expression network construction and key Gene Mining in specific modules

**Fig. 9:**
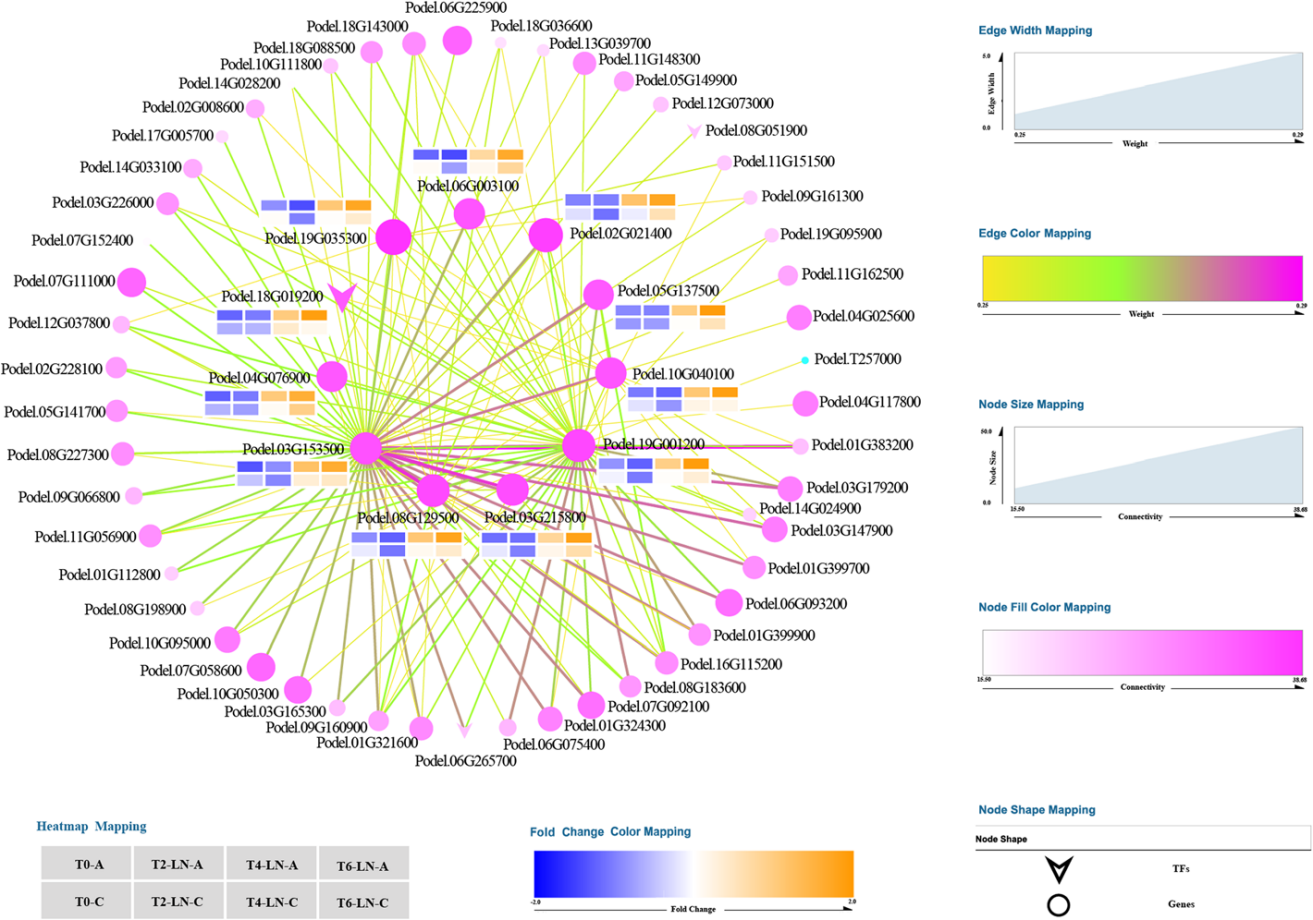
Cytoscape representation of the top 150 network relationships related to hub genes that were selected according to the weight value in the ‘magenta’ module. The color of the lines between genes from orange to green to pink indicates that the correlation (weight value) between genes is becoming stronger, and the thicker the lines, the stronger the correlation (weight value). The larger the node, the pinker the color, indicating the greater connectivity of the gene in the module, and TFs represent transcription factors. The heat map next to the central gene shows the expression level of the gene in different samples, and the color from blue to orange indicates that the expression level is increasing. In the upper row, the four samples from left to right are T0-A, T2-LN-A, T4-LN-A, and T6-LN-A, respectively, and in the next row are T0-C, T2-LN-C, T4-LN-C, and T6-LN-C, respectively

Based on the correlation of 171 genes in the ‘magenta’ module (Table S4), the top 10 genes in terms of connectivity (within the module) were selected as hub genes, and the gene pairs related to the hub genes with the top 150 weight values were selected to construct the network graph (Fig. 9). The 10 genes (except one TF gene, Podel.18G019200) in the center of the network were hub genes, including genes encoding domain-containing proteins [DUF668 domain-containing protein/DUF3475 domain-containing protein (Podel.02G021400), MACPF domain-containing protein At4g24290-like (Podel.19G001200), ACT domain-containing protein ACR4 isoform X1 (Podel.08G129500), C2 and GRAM domain-containing protein At1g03370-like (Podel.06G003100)], echinoderm microtubule-associated protein-like 6 (Podel.19G035300), ABC transporter C family member 3-like (Podel.03G215800), probable serine/threonine-protein kinase clkA (Podel.03G153500), plant intracellular Ras-group-related LRR protein 3-like (Podel.10G040100), UDP-glucoronosyl/UDP-glucosyl transferase family protein UDP (Podel.04G076900), and random slug protein 5-like (Podel.05G137500). Among them, Podel.03G153500 had the strongest connectivity with all other genes and participates in the plant response to stress (Table S5).

The expression levels of most hub genes in the leaves of genotypes A and C decreased slightly in the early stage of low N stress (from T0 to T2), and increased gradually from T2 to T6. Moreover, the expression levels of hub genes in genotype A were lower than those in genotype C at T0, but it was the opposite at T6, indicating more obvious changes in the expressions of hub genes in genotype A (Fig. 9, Table S4).

To further explore the key genes in the ‘magenta’ module, TF annotation was carried out. Finally, 12 TFs were annotated (Table S6), including WRKY TF family genes [*PodelWRKY41* (Podel.01G096500), *PodelWRKY75* (Podel.15G104200), and *PodelWRKY18* (Podel.18G019200)], bHLHTF family genes [*PodelBHLH25* (Podel.01G303700), *PodelBHLH30* (Podel.02G115300), and *PodelBHLH* (Podel.06G162000)], LOB TF family genes [*PodelLBD37* (Podel.07G061600) and *PodelLBD1* (Podel.08G051900)], ARR-B TF family genes [*PodelWER* (Podel.12G083500)], bZIP TF family genes [*PodelHY5* (Podel.06G265700)], SBP TF family genes [*PodelSPL4* (Podel.01G421600)], and zf-HD TF family genes [*PodelZHD4* (Podel.12G042700)]. Among them, the connectivity between *PodelWRKY18* and other genes in the ‘magenta’ module was second only to the hub genes, and the changing trend of its expression with the duration of low N stress was consistent with that of the hub genes (Fig. 9, Table S6).

### Difference in gene expression related to N metabolism under N stress

The expression levels of genes related to N uptake, transport, and assimilation in genotypes A and C during LN treatment were detected using quantitative real-time reverse transcription PCR (qRT-PCR), and the accuracy of transcriptome data was verified by comparing the results of qRT-PCR and RNA sequencing (Fig. S12). Under LN treatment, the expression levels of nitrate (NO_3_^−^) transport related genes [*NRT1;1* (Podel.03G118100) and *NRT1;2* (Podel.12G073800)] were inhibited, and the change trends in genotypes A and C were similar. The expression of *AMT1;6* (Podel.09G046000) was inhibited in genotype A and promoted in genotype C, while the expression of *AMT2;1* (Podel.06G111800) showed the reverse results. In genotype A, the expression levels of *NR* (Podel.05G183500) and *NiR* (Podel.04G146000) decreased at first (from T0 to T4) and then increased (from T4 to T6). In genotype C, these genes were inhibited all the time, but promoted in at T2 and T6. In genotypes A and C, the expression of *GS2* (Podel.10G024300) was upregulated at T2 and gradually decreased from T2 to T6, to a point that was lower than that at T0, and the inhibition in genotype A was stronger than that in genotype C. The expression of *GDH2* (Podel.15G117500) decreased from T0 to T2 and then gradually increased from T2 to T6 in genotype A, with the expression at T6 being higher than that at T0. In genotype C, the expression decreased from T0 to T6.

## Discussion

Although studies on plant responses to low N stress have been carried out at morphological, physiological, and transcriptional levels, most of them were limited to root tissues of a single genotype, such as in maize [37], rice [38], and poplar [39]. However, different genotypes show different tolerances to biotic and abiotic stresses, including low N tolerance [36, 40]. Comparing the differences between two contrasting genotypes for a specific trait can effectively analyze the regulatory relationship between genes, which is widely used to analyze the molecular mechanism of excellent traits and to identify key regulatory genes. Wang et al. revealed that certain mRNAs and miRNAs were differentially expressed between N stress-insensitive (*Nanlin 1388*) and N stress-sensitive (*Nanlin 895*) poplar clones under low N stress, and noted that miRNAs play an important role in plant adaptation to low N [41]. These studies confined themselves to studying a single point in time, whereas in the present study, we explored the morphological differences of two contrasting genotypes of NUE under low N stress and carried out a detailed time-course analysis of enzyme activities and gene expression related to N metabolism in leaves.

### Differences of morphological responses to low N stress

We believe that N-efficient plants have two characteristics, first, they have a stronger N absorption capacity than other plants, which is closely related to the growth state and architecture of the root system; and second, they can efficiently transform the absorbed organic or inorganic N into dry matter through biological processes such as assimilation, which mainly depends on the activities of enzymes related to N metabolism in the roots and leaves, and the growth status of the leaves [42]. Previous studies found that N-efficient genotypes had a more developed root architecture and larger leaf area than N-inefficient genotypes under low N stress, and low N stress stimulated the growth of roots, especially fine roots, in plants with high or low NUE [3, 26, 43]. In our study, the growth of the N-efficient (A-1, A-2, and A-3) and N-inefficient (C-1, C-2, and C-3) genotypes was inhibited under low N stress (Fig. 1). We found that compared with the N-inefficient genotypes, the growth of the N-efficient genotypes was less affected and showed higher tolerance to low N stress (Table S1). Notably, after 40 days of low N stress treatment, the RFW of the N-efficient genotypes increased, while the RDW decreased, indicating that the root architecture experienced adaptive changes, the growth of fine roots increased, and the root absorption capacity was enhanced. Meanwhile, the root growth of the N-inefficient genotypes was significantly inhibited, which affected their root absorption capacity. The leaf growth and morphology-related traits could be used as reliable indicators to evaluate plant NUE [44]. In this study, we found that the leaf area of the N-efficient genotypes was larger than that of the N-inefficient genotypes under CK or LN treatment (Fig. 2), which enhanced the plants’ ability to produce dry matter.

### Differences in the physiological responses to low N stress

As the main component of photosynthetic pigments, in a growing environment, the N content and its availability in leaves affects the synthesis of photosynthetic pigments [9]. These pigments play an important role in the electron transfer process of leaf photosynthesis, and the intensity of photosynthesis will affect the assimilation efficiency of nutrients and the yield of plants. Our study found that the leaves of both genotypes turned yellow after 40 days of low N stress, and measurement of the chlorophyll content showed that low N stress inhibited its synthesis. In addition, under CK or LN treatment, the leaf chlorophyll content of the N-efficient genotypes was higher than that of the N-inefficient genotypes, and the leaf area of the N-efficient genotypes was larger than that of the N-inefficient genotypes, indicating that the N-efficient genotypes, with a higher NutE, could synthesize more carbon and N compounds (Fig. 2).

N metabolism in plants can be summarized as absorption, transport, assimilation, and utilization. Plants absorb inorganic N (NH_4_^+^ and NO_3_^−^) from the soil with the help of transporters on the surface of the roots, and some of the NH_4_^+^ and NO_3_^−^ are assimilated in the roots, while the other part of the NH_4_^+^ and most of the NO_3_^−^, including part of the organic N, is transported to the leaves for assimilation and utilization. During assimilation, NO_3_^−^ is transformed into NH_4_^+^ under the action of NR and NiR. Then, NH_4_^+^ participates in the synthesis of glutamic acid (Glu) under the catalysis of GS and GOGAT. Alternatively, NH_4_^+^ and 2-oxoglutarate directly synthesize Glu with the help of GDH. Glu can be further involved in the synthesis of organic substances that are necessary for plant growth [18, 33]. NR, GS, GOGAT, and GDH play important roles in N metabolism and their activities in the leaves of the N-efficient and N-inefficient genotypes were inhibited under low N stress. The decrease of NR and GDH activities in leaves of the N-efficient genotypes might be related to the decrease of NO_3_^−^ and NH_4_^+^ contents in leaves at the early stage of low N stress, respectively. With the increase of fine root growth, the absorption capacity of roots was enhanced, and the contents of NO_3_^−^ and NH_4_^+^ increased. To maintain the stability of NO_3_^−^ in plants, NR activity increased gradually, resulting in further increase of NH_4_^+^ content, after which the GDH activity increased gradually. However, the activities of GS and GOGAT increased briefly during low N stress, and then decreased thereafter, which indicated that GDH, GS, and GOGAT play an important role in maintaining the balance of NH_4_^+^ in plants [45]. The difference was that the NR activity was low in the leaves of the N-inefficient genotypes, and GDH activity decreased gradually with increasing stress treatment time, which indicated that the N assimilation ability in the leaves of the N-inefficient genotypes was lower than that of the N-efficient genotypes (Fig. S3).

### Differences in transcriptional responses to low N stress and the identification of key genes

In response to low N stress, there are many DEGs among plants with different NUE values, which indicates that the molecular mechanisms of their adaptation are different, resulting in different N absorption and assimilation capacities [26, 46]. In the present study, the RNA-seq results showed that the responses to low N stress of the N-efficient and N-inefficient *P. deltoides* genotypes were different, and a total of 4906 DEGs were detected at four-time points, among which 3055 DEGs were detected at 40 days of treatment (Fig. S5). The genes were enriched in indole alkaloid biosynthesis and plant hormone signal transduction (Fig. 3), indicating that phytohormones play an important role in the response of plants to low N stress [46–48]. Meanwhile, we found that compared with the N-efficient genotypes, the N-inefficient genotypes were more sensitive to low-N stress; however, fewer DEGs (2044) were detected in the process of N starvation (Fig. S7 and 8). Most of the DEGs detected in all genotypes were enriched in metabolic pathways and the biosynthesis of secondary metabolites, which are related to the plant response to abiotic stress [3]. More genes related to plant hormone signal transduction and ABC transporters, which are responsible for taking up inorganic N [8], were specifically detected in the N-efficient genotypes (Fig. 5). Moreover, more genes related to plant-pathogen interactions were found in the leaves of the N-efficient genotypes in response to N starvation (Fig. 6). The differences in the expression levels of genes involved in the response to N starvation in leaves between the genotypes were closely related to their different N uptake, transport, and assimilation capacities.

To date, key genes closely related to NUE have been found in many plants, such as maize [8], rice [24, 49, 50], oilseed rape [3], and poplar [32, 48]. Dash et al. [29, 30] found that three key genes (*PtaHWS*, *PtaNAC1*, and *PtaRAP2.11*) were highly expressed in the poplar (*P. tremula* × *P. alba*) root system under low N stress, which promoted the growth of plant roots and improved the NUE. In our study, we found that the ‘magenta’ module, including 171 genes that are mainly involved in the response to stress and negative regulation of many biological processes, was negatively related to the changing trend of GS, GOGAT, and AAs in the leaves of poplar during the response to low N stress (Figs. 7 and 8). Meanwhile, 10 hub genes and 12 TFs in the ‘magenta’ module might play an important role in the plant response to low N stress (Fig. 9, Table S4). Among the 10 hub genes, Podel.19G001200, which encodes a protein with a membrane attack complex component/perforin (MACPF) domain [51], and Podel.03G153500, which probably encodes a serine/threonine-protein kinase with a development and cell death (DCD) domain [52], are involved in cell development and programmed cell death. The domain of unknown function 668 (DUF668)-containing protein (Podel.02G021400) [53], the GRAM (from glucosyltransferases, Rab-like GTPase activators and myotubularins) domain-containing protein (Podel.06G003100) [54], and UDP-glycosyltransferases (Podel.04G076900) [55] are essential for plant defense against abiotic stress. Previous studies have shown that NUE and signaling pathways are regulated by TFs in response to N starvation [8, 56–58]. Among the TFs found in this study, WRKY TFs, regulating a variety of hormone signaling pathways [59]; bHLH TFs, one of the largest TF families in plants, regulating plant growth and signal transduction [60]; and bZIP TFs, regulating processes including pathway defense, light, and stress signaling [61], were detected in response to low N stress. These TFs were identified as being responsive to N availability in maize [8], *Arabidopsis* [57], and rice [58]. Moreover, the *PodelWRKY18* (Podel.18G019200) was specifically upregulated in N-efficient and N-inefficient genotypes under low N stress, which was consistent with previously published results [57].

### Combination of phenotype data and transcriptome data

Under low N conditions, the genes related to the activity of chlorophyll catabolite reductase (Podel.07G050500, and Podel.07G050700) were upregulated in the leaves of the N-efficient and N-inefficient genotypes, which would promote the decomposition of chlorophyll and reduce the chlorophyll content (Fig. 4). N could be transferred in plants, thus when the absorbed N is insufficient, the N accumulated in chlorophyll is degraded for other growth processes. The transcriptome data showed that the expression of genes (Podel.08G203700, Podel.08G207400, and Podel.10G049300) related to crassulacean acid metabolism (CAM) increased gradually during low N stress in the N-efficient genotypes, and their expression levels were higher than those in the N-inefficient genotypes in the post-processing stage of low N treatment (Fig. 4), indicating that the N-efficient genotypes fixed more carbon for growth than the N-inefficient genotypes. Moreover, some genes related to amino acid biosynthesis in the N-efficient genotypes were gradually overexpressed in the process of low N stress, and the expression levels were higher than those in the N-inefficient genotypes, which would strengthen their amino acid synthesis ability; however, the AA content in leaves may be reduced because of insufficient N supply. In addition, the expression changes of DEGs in the N-efficient genotypes were more obvious than those in the N-inefficient genotypes under low nitrogen stress, which indicated that the N-efficient genotype could adapt to low N stress by markedly changing their gene expression levels. As indicated by the phenotypic data, the adaptability of the N-efficient genotypes was better than that of the N-inefficient genotypes under low N conditions.

The expression levels of *NRT1;1* and *NRT1;2* in the leaves of the two contrasting genotypes were inhibited under low N stress. With increasing treatment time, the expression of *AMT1;6* in the leaves of N-efficient genotypes first increased, then decreased, and finally was lower than that before treatment, and this change trend was the completely opposite to that of *AMT2;1*. In contrast, the expression of *AMT1;6* in the leaves of the N-inefficient genotypes was upregulated and that of *AMT2;1* was downregulated under low N conditions. The results indicated that there were differences in the absorption and transport mechanism of NH_4_^+^ between the N-efficient and N-inefficient genotypes. The results of qRT-PCR or RNA-seq of N assimilation-related genes (*NR*, *GS2*, and *GDH2*, Fig. S3) in plant leaves support the changing trend of related enzyme activities (NR, GS, and GDH, Fig. S3). Moreover, we speculated that the upregulation of *AMT2;1* in the leaves of the N-efficient genotypes could inhibit *GS2* expression and induce *GDH2* expression, which is of great significance in maintaining the stability of the NH_4_^+^ content.

In summary, there were significant differences in the molecular mechanisms between the N-efficient and N-inefficient genotypes in response to low N stress. To the best of our knowledge, this is the first time-course analysis of enzyme activities and gene expression related to N metabolism in leaves of two contrasting genotypes under low N stress. The results could provide valuable information to understand the efficient N assimilation and utilization capacity of *P. deltoides*.

## Conclusions

In the present study, plant growth, chlorophyll synthesis, and enzyme activities related to N metabolism of *P. deltoides* were inhibited under low N stress, and the N-efficient genotypes showed stronger adaptability and a better NUE than the N-inefficient genotypes. The time-course analysis of transcriptome data revealed that compared with the N-inefficient genotypes, more genes related to N assimilation and plant hormone signal transduction were involved in the response to low N stress in the leaves of N-efficient genotypes, and the sensitivity to N starvation was weak. Under low N stress, the upregulated expression of genes related to the negative regulation of the life process in leaves slowed down the life activity of plants and enhanced their defensive ability to cope with N starvation. The discovery of hub genes related to programmed cell death and the defense response, and TFs related to signal transduction, might provide a valuable theoretical basis for analyzing the molecular mechanism of efficient N t utilization and improving the NUE of poplar.

## Methods

### Plant materials and treatments

The samples used in this study were collected from the germplasm resource bank of *P. deltoides* in Ningyang, Shandong Province, China (35°55′39″N, 116°53′59″E). We obtained the germplasm resources of *P. deltoides* in the form of gift, through the international exchange and cooperation. This bank was established by our research group using these samples and we are in charge of it. The details of the plant materials have also been clarified in our previous article published in BMC Genetics [35]. Based on previous research results, three N-efficient genotypes (A-1, A-2, and A-3) and three N-inefficient genotypes (C-1, C-2, and C-3) of *P. deltoides* were used in this study [36]. Among them, A-1 and A-3 come from Tennessee and belong to different families; A-2 and C-1 come from Quebec, Canada and belong to the same family; C-2 comes from Louisiana; and C-3 comes from Iowa. One-year-old cuttings (15 cm in length, 1.5 cm in diameter) of each genotype were rooted and cultured in nutritional pots (5 cm in height, 5 cm in caliber) filled with medium (nutrient soil:perlite = 9:1). After cultivation for 40 days in a greenhouse at the Chinese Academy of Forestry (40°0′10″ N, 116°14′38″ E), 40 uniform plants of each genotype were selected and the root systems of the plants were carefully washed with running water. The plants were then cultured with water in new pots (20 cm in height, 10 cm in caliber) filled with vermiculite for 15 days. Subsequently, the plants were irrigated every other day with 100 ml of one-tenth strength Hoagland nutrient solution, which contained 0.5 mM KNO_3_, 0.4 mM Ca (NO_3_)_2_·4H_2_O, 0.4 mM MgSO_4_·7H_2_O, 0.1 mM NH_4_NO_3_, 0.1 mM KH_2_PO_4_, 5 μM Fe-EDTA (pH = 5.5), 50 nM H_3_BO_3_, 50 nM MnSO_4_·4H_2_O, 15 nM ZnSO_4_·7H_2_O, 2.5 nM KI, 0.5 nM Na_2_MoO_4_·2H_2_O, 0.05 nM CuSO_4_·5H_2_O, and 0.05 nM CoCl_2_. The solution was adjusted to pH 6.0. After 20 days, 30 plants from each genotype were selected and randomly divided into two groups (15 plants in each group) for N treatment. Plants of the two groups were cultivated with modified Hoagland nutrient solution (0.5 mM KCl, 0.4 mM CaCl_2_·2H_2_O, 0.4 mM MgSO_4_·7H_2_O, 0.1 mM KH_2_PO_4_, 5 μM Fe-EDTA [pH = 5.5], 50 nM H_3_BO_3_, 50 nM MnSO_4_·4H_2_O, 15 nM ZnSO_4_·7H_2_O,2.5 nM KI, 0.5 nM Na_2_MoO_4_·2H_2_O, 0.05 nM CuSO_4_·5H_2_O, and 0.05 nM CoCl_2_) containing 5 μM (LN) or 750 μM (CK) NH_4_NO_3_, respectively, every 2 days. The N treatments were maintained for 40 days before harvest.

### Sample collection

The leaves of each genotype were sampled at 0 (T0), 3 (T1), 5 (T2), 10 (T3), 20 (T4), 30 (T5), and 40 (T6) days of N-treatment. Three biological repeats were taken for each genotype in the LN or CK treatment, and each replicate included 2–3 mature functional leaves. The sampling time was between 9:00 am and 10:00 am. A mixed leaf sample was obtained by mixing one biological repeat leaf sample of the three N-efficient genotypes or N-inefficient genotypes at the same time point in the same N treatment. Finally, at each time point of each treatment, there were three mixed samples for the two NUE type plants, respectively. These mixed samples were stored at -80 °C for the determination of enzyme activities related to N metabolism and for transcriptome sequencing.

### Enzyme activity assay

The activities of nitrate reductase (NR, EC 1.7.1.3), glutamine synthetase (GS, EC 6.3.1.2), glutamine oxoglutarate aminotransferase (GOGAT, EC 1.4.7.1), and glutamate dehydrogenase (GDH, EC 1.4.1.2) in the mixed leaf samples were detected using the relevant biochemical kits (BC0085, BC0915, BC0075, and BC1465, respectively, Solarbio, Beijing, China). The detection steps followed the manufacturer’s instructions strictly.

### Measurement of free amino acids and soluble sugar in leaves

The contents of free amino acids (AAs) and soluble sugars (SSs) in mixed samples of leaves were determined using the corresponding biochemical kits (BC1575 and BC0035, respectively, Solarbio) according to the manufacturer’s protocol.

### RNA extraction, library construction, and sequencing

Total RNA was extracted from leaves using a Trizol reagent kit (Invitrogen, Carlsbad, CA, USA). RNA purity was detected using a Nanodrop 2000 microspectrophotometer (Thermo Fisher, Waltham, MA, USA), and the RNA quality was assessed on an Agilent 2100 Bioanalyzer (Agilent Technologies, Santa Clara, CA, USA) and checked using RNase free agarose gel electrophoresis. Sequencing libraries were constructed using a NEBNext Ultra RNA Library Prep Kit for Illumina (#E7530, NEB, Ipswich, MA, USA) according to the manufacturer’s instructions. Sequencing was performed using Illumina NovaSeq 6000 (Illumina, San Diego, CA, USA) by Gene Denovo Biotechnology Co. (Guangzhou, China).

### RNA sequencing data analysis

The raw reads were filtered using fastp v0.18.0 [62] to obtain high-quality clean reads, and the clean reads were mapped to the reference genome of *P. deltoides* (JGI 2.1) using HISAT2. 2.4 [63]. The mapped reads of each mixed sample were assembled using StringTie v1.3.1 [64, 65]. For each transcription region, the FPKM (fragment per kilobase of transcript per million mapped reads) value was calculated to quantify its expression abundance using StringTie software. DEGs between two different samples were screened with the parameters of a false discovery rate (FDR) < 0.05 and absolute fold change > 2 using DESeq2 software [66]. First, the differences in the expression of mixed samples between N-efficient and N-inefficient genotypes were compared at the same time point in the LN treatment. Then, the differences in gene expression of N-efficient or N-inefficient genotypes at different time points during LN treatment were studied. All DEGs were mapped to GO terms in the database: http://www.geneontology.org/ [67]. A calculated *p*-value < 0.05 defined a significantly enriched GO term for the DEGs. Pathway enrichment analysis was performed to test the statistical enrichment of DEGs in the KEGG pathways [68]. KEGG pathways with a corrected *p*-value < 0.05 were considered as significantly enriched pathways for the DEGs. WGCNA was performed using the ‘WGCNA (v1.47)’ package in R to find modules of highly correlated genes and to relate the modules to specific traits [69]. The relationship network between selected DEGs was visualized with the help of Cytoscape (v 3.7.1) software [70]. Bioinformatic analysis of transcriptome data was performed using Omicsmart, a real-time interactive online platform for data analysis (http://www.omicsmart.com).

### Quantitative real-time reverse transcription PCR analysis

We conducted qRT-PCR for genes related to N metabolism (*NRT1;1*, *NRT1;2*, *AMT1;6*, *AMT2;1*, *NR*, *NiR*, *GS2*, and *GDH2*) using total RNA extracted from three biological repeats of mixed samples. The TB Green Premix Ex Taq II (Takara, Dalian, China) was used to perform the quantitative real-time PCR step of qRT-PCR in a LightCycler 480 Instrument II system (Roche, Basel, Switzerland). The PCR conditions were as follows: 95 °C for 30 s; 40 cycles of 95 °C for 5 s and 60 °C for 30 s; followed by 95 °C for 5 s and 60 °C for 1 min. The reaction system is listed in Table S7. *Actin 2/7* was used as an internal reference, and relative expression levels were calculated using the 2^-ΔΔCt^ method [71]. The gene-specific primer pairs are listed in Table S8. The accuracy of transcriptome sequencing data was tested based on the results of qRT-PCR.

### Determination of leaf morphological characteristics and plant biomass

The height and the ground diameter of each plant were determined before the N treatments (H0 and GD0) and at harvest (Hn and GDn), respectively. After N treatment (40 days), three plants whose height was similar to the mean height were selected for each genotype in each treatment. Three to five mature and complete functional leaves per plant were obtained, and the leaf morphological characteristics (leaf length, leaf width, and leaf area) were measured using a leaf area meter (Yaxin-1241, Beijing Yaxin Liyi Technology Co., Ltd., Beijing, China). The root system of each plant was carefully washed and collected. The fresh weights of the stem, root, and leaves of each selected plant were recorded. Subsequently, they were dried at 75 °C for 96 h until their weights were constant, and then their dry weights were recorded. Finally, to facilitate comparative analysis, the data related to the leaves were transformed into single leaf data.

### Measurement of leaf chlorophyll

After N treatment, the concentrations of chlorophyll in the leaves were measured using the 96% ethanol method [72], and a total of three biological repeats of each genotype were tested in the LN or CK groups. The method was as follows: A leaf sample (0.1 g) was accurately weighed and put into a 5 ml centrifuge tube, and 4 ml of 96% ethanol was added to extract chlorophyll from the leaves. After the chlorophyll in the leaves was completely extracted (about 6 h in darkness), the absorbance values at 470 nm, 649 nm, and 665 nm were measured using a Molecular Devices spectrophotometer (Thermo Fisher, Waltham, MA, USA). The concentration of chlorophyll in the extract was calculated using formulae 1–4.

1 $$ {\mathrm{C}}_{\mathrm{a}}\left(\mathrm{mg}.{\mathrm{L}}^{-1}\right)=13.95{A}_{665}-6.88{A}_{649} $$

2 $$ {\mathrm{C}}_{\mathrm{b}}\left(\mathrm{mg}.{\mathrm{L}}^{-1}\right)=24.69{A}_{649}-7.32{A}_{665} $$

3 $$ \mathrm{Car}\left(\mathrm{mg}.{\mathrm{L}}^{-1}\right)=\left(1000{A}_{470}-2.05{\mathrm{C}}_{\mathrm{a}}-114.8{\mathrm{C}}_{\mathrm{b}}\right)/245 $$

4 $$ {\mathrm{C}}_{\mathrm{T}}\left(\mathrm{mg}.{\mathrm{L}}^{-1}\right)={\mathrm{C}}_{\mathrm{a}}+{\mathrm{C}}_{\mathrm{b}} $$

Where *A*_470_, *A*_649,_ and *A*_665_ represent the absorbance values at 470, 649, and 665 nm, respectively; and C_a_, C_b_, Car, and C_T_ represent the concentrations of chlorophyll a, chlorophyll b, carotenoids, and total chlorophyll in the extract, respectively.

The content of chlorophyll in leaves was calculated using formula 5.

5 $$ \mathrm{Chl}\left(\mathrm{mg}.{\mathrm{g}}^{-1}\mathrm{FW}\right)=\mathrm{C}\left(\mathrm{mg}.{\mathrm{L}}^{-1}\right)\times \mathrm{V}\left(\mathrm{L}\right)/\mathrm{m}\left(\mathrm{g}\right) $$

Where C is the concentration of chlorophyll; V is the volume of the extract; m is the fresh weight of the tested leaves; Chl is the amount of chlorophyll (mg) in 1 g fresh leaves. Chl a, Chl b, Car, and Chl are used to represent the contents of chlorophyll a, chlorophyll b, carotenoids, and total chlorophyll, respectively.

### Statistical analyses

All experiments performed in this study were performed using three biological repeats and three experimental repeats. The phenotypic, physiological, and qRT-PCR data were recorded using Microsoft Excel (Microsoft Corporation, Redmond, WA, USA), and the mean and standard deviation (SD) values of every parameter of each genotype in LN or CK treatment were calculated. The ratio of the measured values of characteristics under LN treatment and that under CK treatment was used as the LNAC of the plants, which was used to evaluate the adaptability of plant traits to low N conditions. Differences between different comparison groups were determined using one-way analysis of variance (ANOVA) concatenated with Duncan test (*p* < 0.05) using the package ‘agricolae’ in R (v 3.5.3).

## Supplementary Information

**Additional file 1: Fig. S1.** Effects of low N stress on the growth traits, leaf morphology, and chlorophyll content of N-efficient (A-1, A-2, and A-3) and N-inefficient (C-1, C-2, and C-3) genotypes. Different letters above the columns indicate significant differences between groups (*p* < 0.05). (A) Dry weight of the stem (SDW); (B) Dry weight of the root (RDW); (C) Dry weight of the leaf (LDW); (D) Chlorophyll a (Chl a); (E) Chlorophyll b (Chl b); (F) Carotenoid (Car); (G) Leaf length (LL); (H) Leaf width (LW). **Fig. S2. **Morphologies of the leaves (a-f) and roots roots (g-l) morphological photos of the N-efficient (a-c, g-i) and N-inefficient (d-f, j-l) genotypes responding to N limitation. N41: A-1, 141: A-2, N49: A-3; 180: C-1, N16: C-2, 5009: C-3. **Fig. S3.** The change trends of enzyme activities, total amino acid contents, and soluble sugar contents in leaves during N treatment of N-efficient (A) and N-inefficient (C) genotypes. T0, T1, T2, T3, T4, T5, and T6 represent 0, 3, 5, 10, 20, 30, and 40 days of N treatment, respectively. “*” indicates significant differences between LN and CK treatments in the A or C genotypes (*p* < 0.05). (A) nitrate reductase activities (NR); (B) glutamine synthetase activities (GS); (C) glutamate dehydrogenase activities (GDH); (D) glutamine oxoglutarate aminotransferase (GOGAT); (E) Total amino acid contents (AAs); (F) Soluble sugar contents (SSs). **Fig. S4.** Transcriptome relationships among three biological replicates. A: N-efficient genotypes; C: N-inefficient genotypes. T0, T2, T4, and T6 represent 0, 5, 20, and 40 days of N treatment, respectively. LN: low N treatment. **Fig. S5.** (A) Bar chart showing numbers of upregulated and downregulated differentially expressed genes (DEGs) in the four comparison groups (T0-C vs. T0-A, T2-LN-C* vs. *T2-LN-A, T4-LN-C vs. T4-LN-A and T6-LN-C vs. T6-LN-A; LN: low nitrogen treatment). The magenta column shows upregulated DEGs, and the cyan column shows downregulated DEGs. (B) Venn diagram showing that the distribution of DEGs identified in the comparison of genotypes A and C are common and specific to T0, T2, T4, and T6. **Fig. S6.** Results of the gene ontology (GO) functional enrichment analysis of the special differentially expressed genes (DEGs) in genotypes A at different time points during the response to low N stress. (a-d) Represent the GO results of the specific DEGs at T0, T2, T4, and T6 in genotypes A, respectively. **Fig. S7.** (A) and (B) bar charts show the numbers of upregulated and downregulated differentially expressed genes (DEGs) in the three comparison groups of A (T0-A vs. T2-LN-A, T2-LN-A *vs. *T4-LN-A, and T4-LN-A vs. T6-LN-A) and C (T0-C vs. T2-LN-C, T2-LN-C vs. T4-LN-C, and T4-LN-C vs. T6-LN-C; LN: low nitrogen) genotypes, respectively. The magenta column shows upregulated DEGs, and the cyan column shows downregulated DEGs. (C) Venn diagrams showing that the distribution of DEGs identified in the comparison of different periods are common and specific to genotypes A and C. DEGs-A and DEGs-C represent all the DEGs identified from genotypes A and C during low N stress treatment, respectively. **Fig. S8.** Top 20 gene ontology (GO) terms of the special and common differentially expressed genes (DEGs) between genotypes A and C during the response to low N stress. (a) Represents the top 20 GO terms of the specific DEGs in genotypes A, (b) represents the top 20 GO terms of the common DEGs in genotypes A and C, and (c) represents the top 20 GO terms of the specific DEGs in genotypes C. **Fig. S9.** Gene expression patterns across four-time points (T0, T2, T4, and T6) in genotypes A and C under low N stress. (A) and (C) indicate the variation trend of differentially expressed genes (DEGs) in genotypes A and C, respectively. Above the box is the ID of the changing trend, and the number in the box indicates the number of DEGs contained in the trend. The grid with color indicates a significantly enrichment trend (*p* < 0.05), and the closer the color is, the more similar the changing trend is. (B) and (D) represent the changing trend of genes in profile 12 with genotype A and profile 17 with genotype C, respectively. **Fig. S10.** Top 20 gene ontology (GO) terms of differentially expressed genes (DEGs) in profile 12 of genotype A (a) and profile 17 of genotype C (b). **Fig. S11.** Expression pattern analysis of genes in the ‘magenta’ module. Red: upregulated; blue: downregulated. A: N-efficient genotypes; C: N-inefficient genotypes. T0, T2, T4, and T6 represent 0, 5, 20, and 40 days of N treatment, respectively. LN: low N treatment. **Fig. S12.** Expression of key genes in nitrogen metabolism in the leaves of genotypes A and C. (A-H) represent the expression trends of *NRT1;1*, *NRT1;2*, *AMT1;6*, *AMT2;1*, *NR*, *NiR*, *GS2,* and *GDH2*, respectively. The columns represent the results of RNA sequencing, and the lines show the qRT-PCR results. Vertical bars indicate SDs (*n* = 3) in the qRT-PCR analysis. A: N-efficient genotypes; C: N-inefficient genotypes.

**Additional file 2: Table S1.** Low nitrogen adaptation coefficients of the traits of N-efficient and N-inefficient genotypes. **Table S2.** Differences in enzyme activities, total amino acid contents, and soluble sugar contents in leaves during N treatment of N-efficient (A) and N-inefficient (C) genotypes. **Table S3.** Results of quality analysis of the RNA sequencing data. **Table S4.** Genes in the ‘magenta’ module. **Table S5.** Annotation description of the top 10 genes for connectivity (hub genes) in the ‘magenta’ module. **Table S6.** Annotation of transcription factors in the ‘magenta’ module. **Table S7.** The reaction system of quantitative real-time reverse transcription PCR (qRT-PCR). **Table S8.** Primers used for quantitative real-time reverse transcription PCR (qRT-PCR) analysis.

## Data Availability

The sequence data reported in this paper have been deposited in the Genome Sequence Archive [73] in National Genomics Data Center [74], Beijing Institute of Genomics (China National Center for Bioinformation), Chinese Academy of Sciences, under accession number CRA003529, which are publicly accessible at https://bigd.big.ac.cn/gsa.

## Abbreviations

N: Nitrogen
A: N-efficient genotypes
C: N-inefficient genotypes
LN: Low nitrogen treatment
CK: Normal nitrogen supply treatment
Hn: Height after treatment
GDn: Ground diameter after treatment
SFW: Fresh weight of the stem
SDW: Dry weight of the stem
RFW: Fresh weight of the root
RDW: Dry weight of the root
LFW: Fresh weight of the leaf
LDW: Dry weight of the leaf
Chl a: Chlorophyll a
Chl b: Chlorophyll b
Car: Carotenoid
Chl: Chlorophyll (a + b)
LL: Leaf length
LW: Leaf width
LA: Leaf area
NR: Nitrate reductase activities
GS: Glutamine synthetase activities
GDH: Glutamate dehydrogenase activities
GOGAT: Glutamine oxoglutarate aminotransferase
AAs: Total amino acid contents
SSs: Soluble sugar contents
DEG: Differentially expressed gene
GO: Gene ontology
KEGG: Kyoto Encyclopedia of Genes and Genomes
WGCNA: Weighted gene co-expression network analysis
