# Supplementary material for: Morphological, physiological, and transcriptional responses to low nitrogen stress in Populus deltoides Marsh. clones with contrasting nitrogen use efficiency
Source: BMC Genomics. 2021 Sep 27;22:697. doi: 10.1186/s12864-021-07991-7 (PMC8474845; doi:10.1186/s12864-021-07991-7)
Supplement: Supplementary file 1 — Additional file 1: Fig. S1. Effects of low N stress on the growth traits, leaf morphology, and chlorophyll content of N-efficient (A-1, A-2, and A-3) and N-inefficient (C-1, C-2, and C-3) genotypes. Different letters above the columns indicate significant differences between groups (p < 0.05). (A) Dry weight of the stem (SDW); (B) Dry weight of the root (RDW); (C) Dry weight of the leaf (LDW); (D) Chlorophyll a (Chl a); (E) Chlorophyll b (Chl b); (F) Carotenoid (Car); (G) Leaf length (LL); (H) Leaf width (LW). Fig. S2. Morphologies of the leaves (a-f) and roots roots (g-l) morphological photos of the N-efficient (a-c, g-i) and N-inefficient (d-f, j-l) genotypes responding to N limitation. N41: A-1, 141: A-2, N49: A-3; 180: C-1, N16: C-2, 5009: C-3. Fig. S3. The change trends of enzyme activities, total amino acid contents, and soluble sugar contents in leaves during N treatment of N-efficient (A) and N-inefficient (C) genotypes. T0, T1, T2, T3, T4, T5, and T6 represent 0, 3, 5, 10, 20, 30, and 40 days of N treatment, respectively. “*” indicates significant differences between LN and CK treatments in the A or C genotypes (p < 0.05). (A) nitrate reductase activities (NR); (B) glutamine synthetase activities (GS); (C) glutamate dehydrogenase activities (GDH); (D) glutamine oxoglutarate aminotransferase (GOGAT); (E) Total amino acid contents (AAs); (F) Soluble sugar contents (SSs). Fig. S4. Transcriptome relationships among three biological replicates. A: N-efficient genotypes; C: N-inefficient genotypes. T0, T2, T4, and T6 represent 0, 5, 20, and 40 days of N treatment, respectively. LN: low N treatment. Fig. S5. (A) Bar chart showing numbers of upregulated and downregulated differentially expressed genes (DEGs) in the four comparison groups (T0-C vs. T0-A, T2-LN-C vs. T2-LN-A, T4-LN-C vs. T4-LN-A and T6-LN-C vs. T6-LN-A; LN: low nitrogen treatment). The magenta column shows upregulated DEGs, and the cyan column shows downregulated DEGs. (B) Venn diagram showing that t [file 12864_2021_7991_MOESM1_ESM.docx]

**Supplementary Figures To**

**Morphological, physiological, and transcriptional responses to low nitrogen stress in *Populus deltoides* Marsh. clones with contrasting nitrogen use efficiency**

Cun Chen ^1, 2 ✝^, Yanguang Chu ^1, 2 ✝^, Qinjun Huang ^1, 2^, Weixi Zhang ^1, 2^, Changjun Ding ^1, 2^, Jing Zhang ^1, 2^, Bo Li ^1, 2^, Tengqian Zhang ^1, 2^, Zhenghong Li ^1, 2^, Xiaohua Su ^1, 2, 3,*^

^1^ State Key Laboratory of Tree Genetics and Breeding, Research Institute of Forestry, Chinese Academy of Forestry.

^2^ Key Laboratory of Tree Breeding and Cultivation, State Forestry and Grassland Administration, Beijing, China.

^3^ Co-Innovation Center for Sustainable Forestry in Southern China, Nanjing Forestry University, Nanjing, Jiangsu Province, China.

✝ These authors contributed equally to this work.

* Correspondence: [suxh@caf.ac.cn](mailto:suxh@caf.ac.cn); Tel.: 86-10-6288-9627

**Figure S1.** Effects of low N stress on the growth traits, leaf morphology, and chlorophyll content of N-efficient (A-1, A-2, and A-3) and N-inefficient (C-1, C-2, and C-3) genotypes. Different letters above the columns indicate significant differences between groups (*p* < 0.05). (A) Dry weight of the stem (SDW); (B) Dry weight of the root (RDW); (C) Dry weight of the leaf (LDW); (D) Chlorophyll a (Chl a); (E) Chlorophyll b (Chl b); (F) Carotenoid (Car); (G) Leaf length (LL); (H) Leaf width (LW).


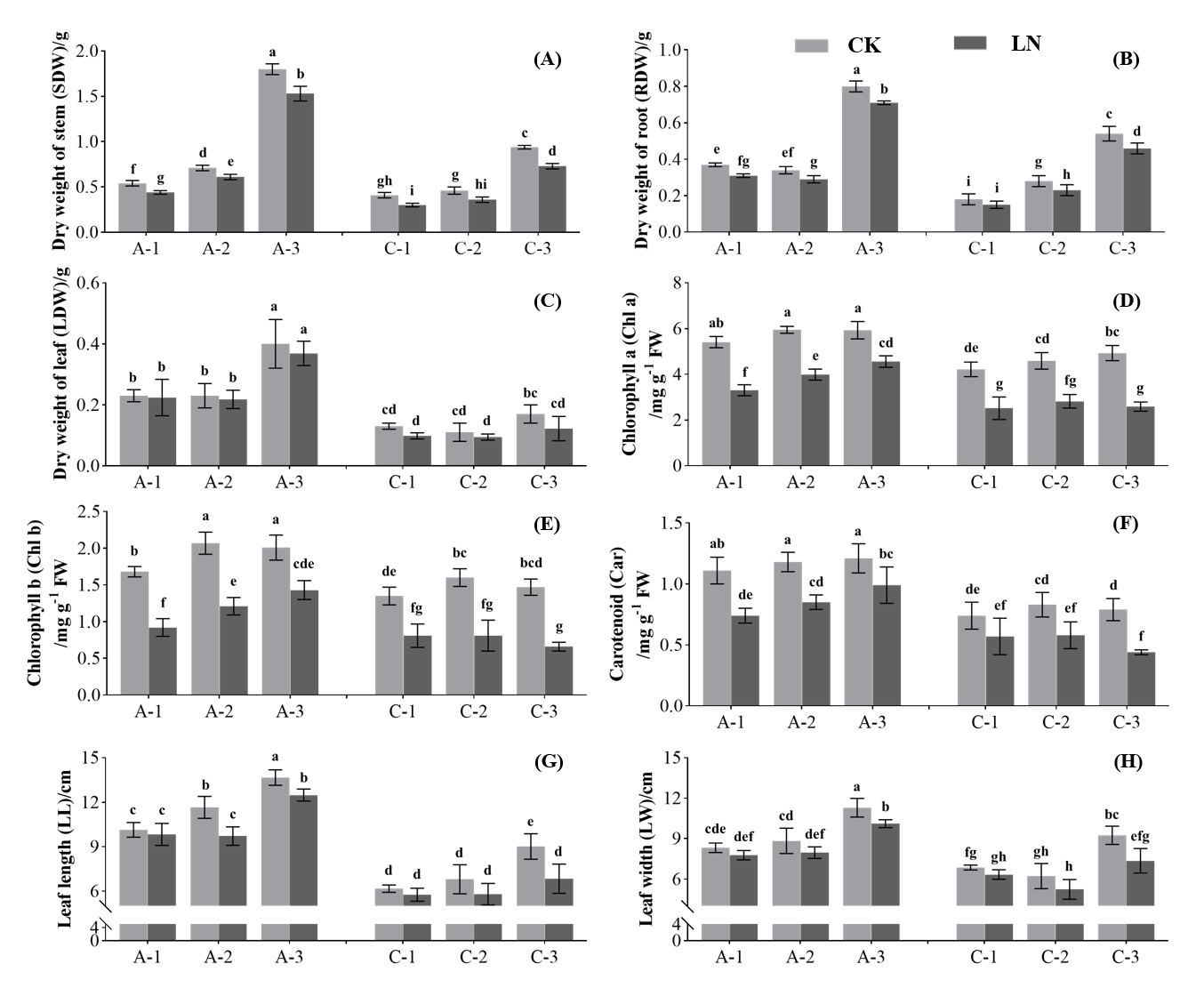


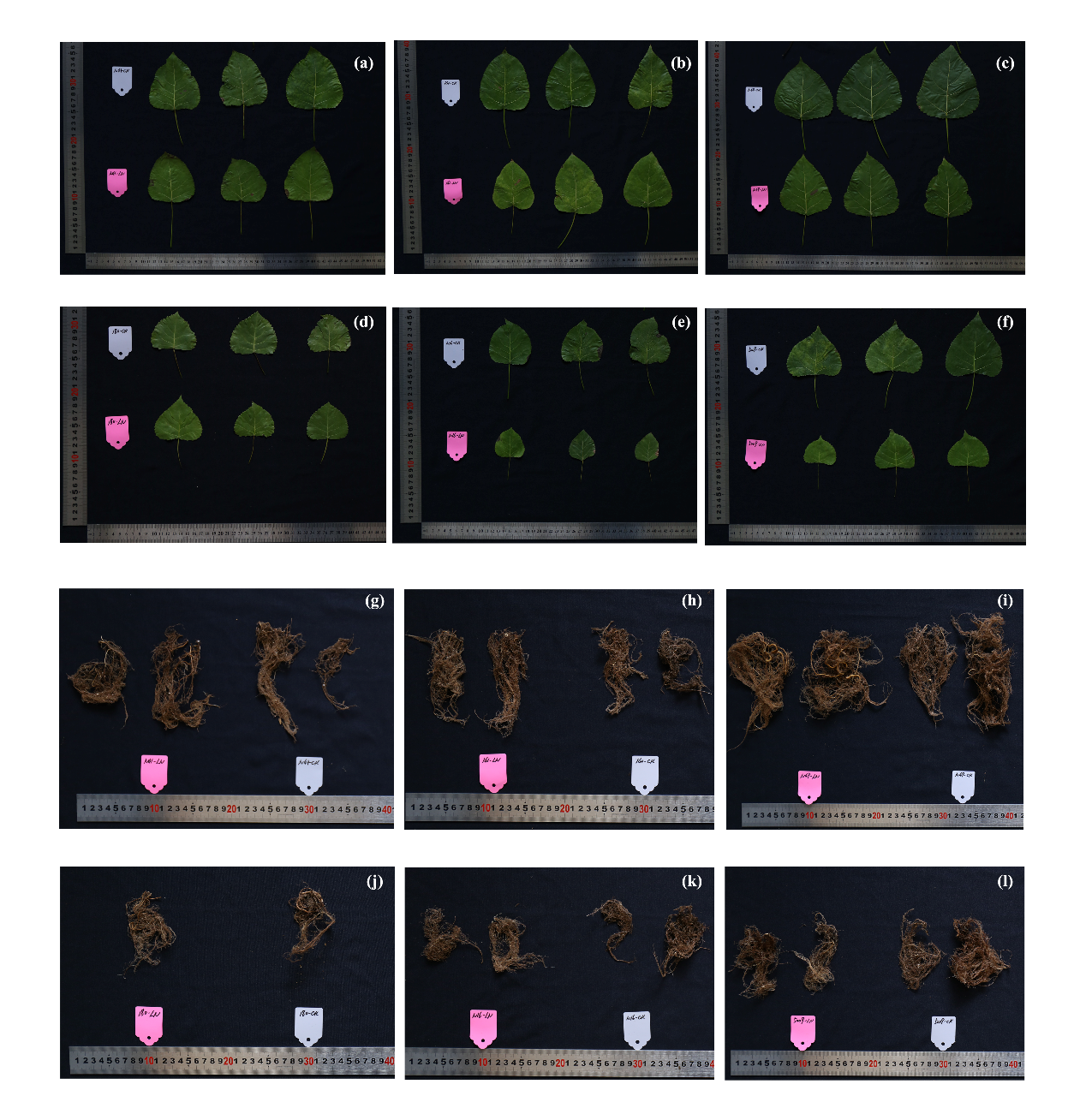


**Figure S2.** Morphologies of the leaves (a-f) and roots roots (g-l) morphological photos onof the N-efficient (a-c, g-i) and N-inefficient (d-f, j-l) genotypes responding to N limitation. N41: A-1, 141: A-2, N49: A-3; 180: C-1, N16: C-2, 5009: C-3.


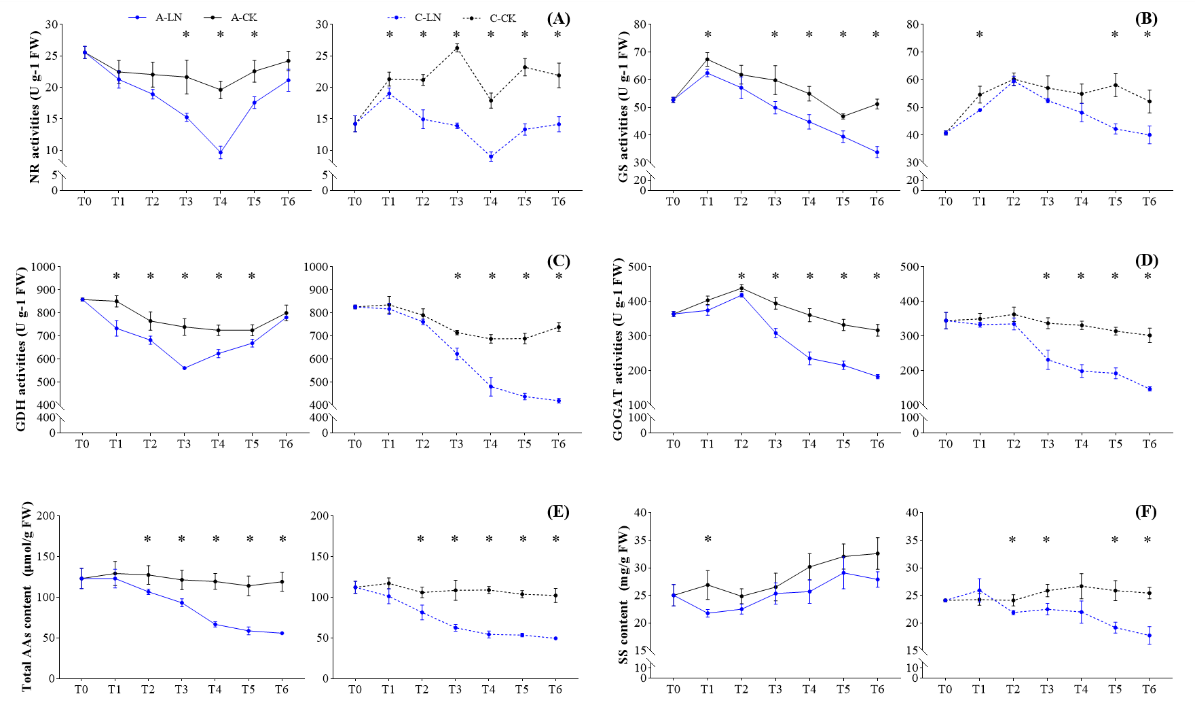


**Figure S3.** The change trends of enzyme activities, total amino acid contents, and soluble sugar contents in leaves during N treatment of N-efficient (A) and N-inefficient (C) genotypes. T0, T1, T2, T3, T4, T5, and T6 represent 0, 3, 5, 10, 20, 30, and 40 days of N treatment, respectively. “*” indicates significant differences between LN and CK treatments in the A or C genotypes (*p* < 0.05). (A) nitrate reductase activities (NR); (B) glutamine synthetase activities (GS); (C) glutamate dehydrogenase activities (GDH); (D) glutamine oxoglutarate aminotransferase (GOGAT); (E) Total amino acid contents (AAs); (F) Soluble sugar contents (SSs).


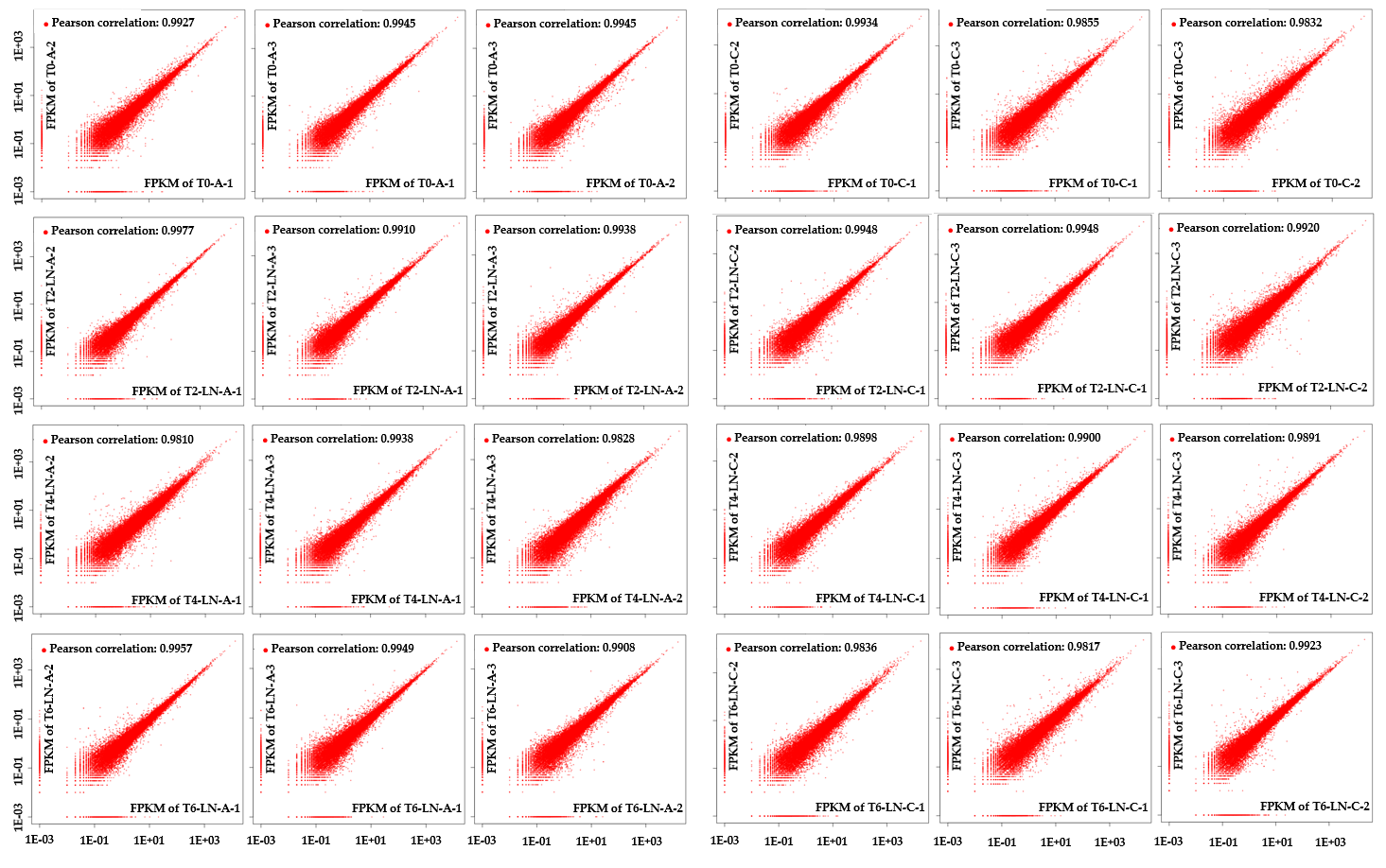


**Figure S4.** Transcriptome relationships among three biological replicates. A: N-efficient genotypes; C: N-inefficient genotypes. T0, T2, T4, and T6 represent 0, 5, 20, and 40 days of N treatment, respectively. LN: low N treatment.


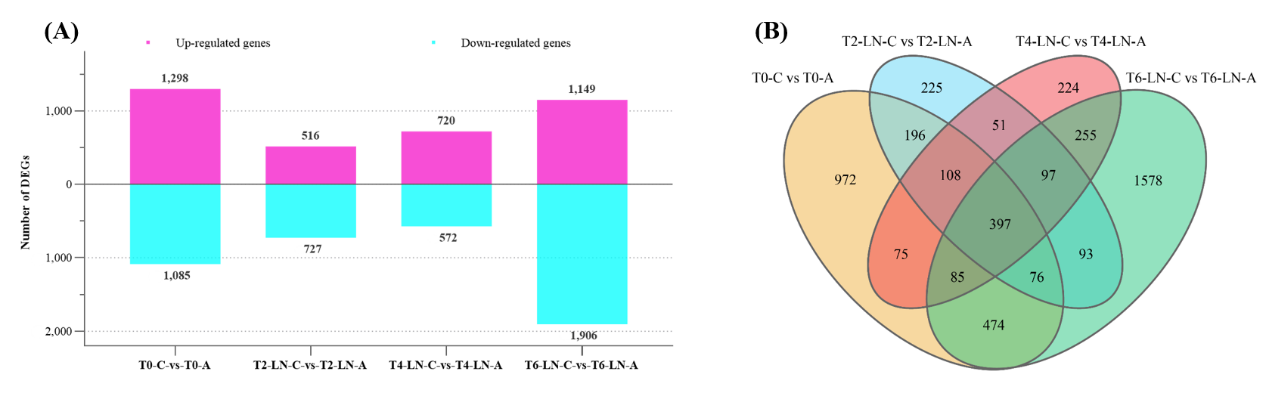


**Figure S5.** (A) Bar chart showing numbers of upregulated and downregulated differentially expressed genes (DEGs) in the four comparison groups (T0-C *vs*. T0-A, T2-LN-C *vs*. T2-LN-A, T4-LN-C *vs*. T4-LN-A and T6-LN-C *vs*. T6-LN-A; LN: low nitrogen treatment). The magenta column shows upregulated DEGs, and the cyan column shows downregulated DEGs. (B) Venn diagram showing that the distribution of DEGs identified in the comparison of genotypes A and C are common and specific to T0, T2, T4, and T6.


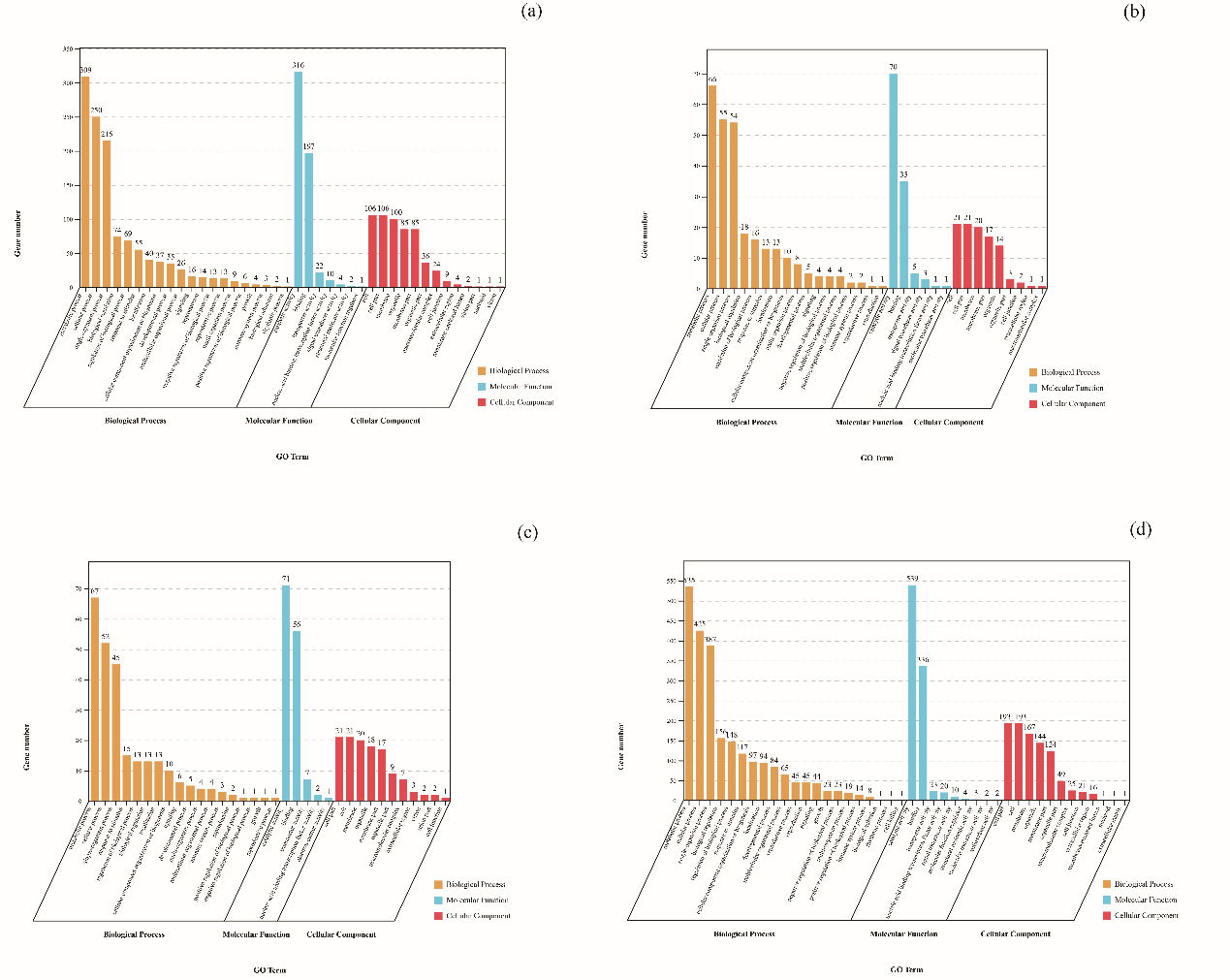


**Figure S6.** Results of the gene ontology (GO) functional enrichment analysis of the special differentially expressed genes (DEGs) in genotypes A at different time points during the response to low N stress. (a-d) Represent the GO results of the specific DEGs at T0, T2, T4, and T6 in genotypes A, respectively.


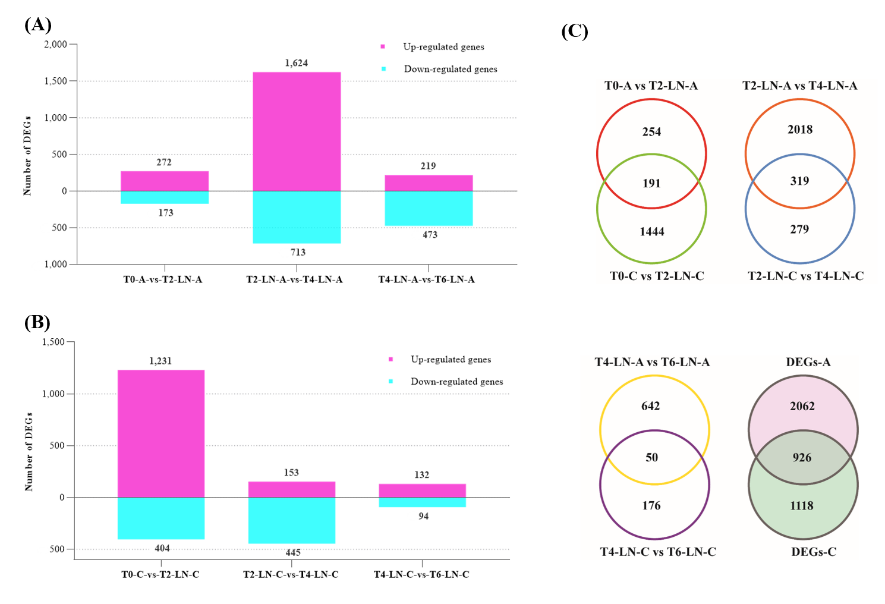
**Figure S7.** (A) and (B) bar charts show the numbers of upregulated and downregulated differentially expressed genes (DEGs) in the three comparison groups of A (T0-A *vs*. T2-LN-A, T2-LN-A *vs*. T4-LN-A, and T4-LN-A *vs*. T6-LN-A) and C (T0-C *vs*. T2-LN-C, T2-LN-C *vs*. T4-LN-C, and T4-LN-C *vs*. T6-LN-C; LN: low nitrogen) genotypes, respectively. The magenta column shows upregulated DEGs, and the cyan column shows downregulated DEGs. (C) Venn diagrams showing that the distribution of DEGs identified in the comparison of different periods are common and specific to genotypes A and C. DEGs-A and DEGs-C represent all the DEGs identified from genotypes A and C during low N stress treatment, respectively.


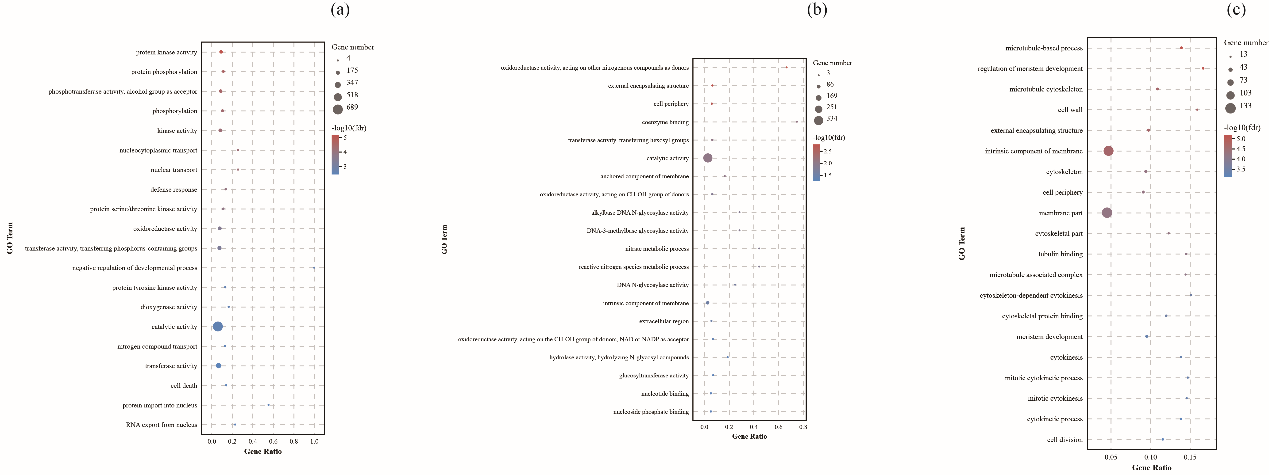


**Figure S8.** Top 20 gene ontology (GO) terms of the special and common differentially expressed genes (DEGs) between genotypes A and C during the response to low N stress. (a) Represents the top 20 GO terms of the specific DEGs in genotypes A, (b) represents the top 20 GO terms of the common DEGs in genotypes A and C, and (c) represents the top 20 GO terms of the specific DEGs in genotypes C.


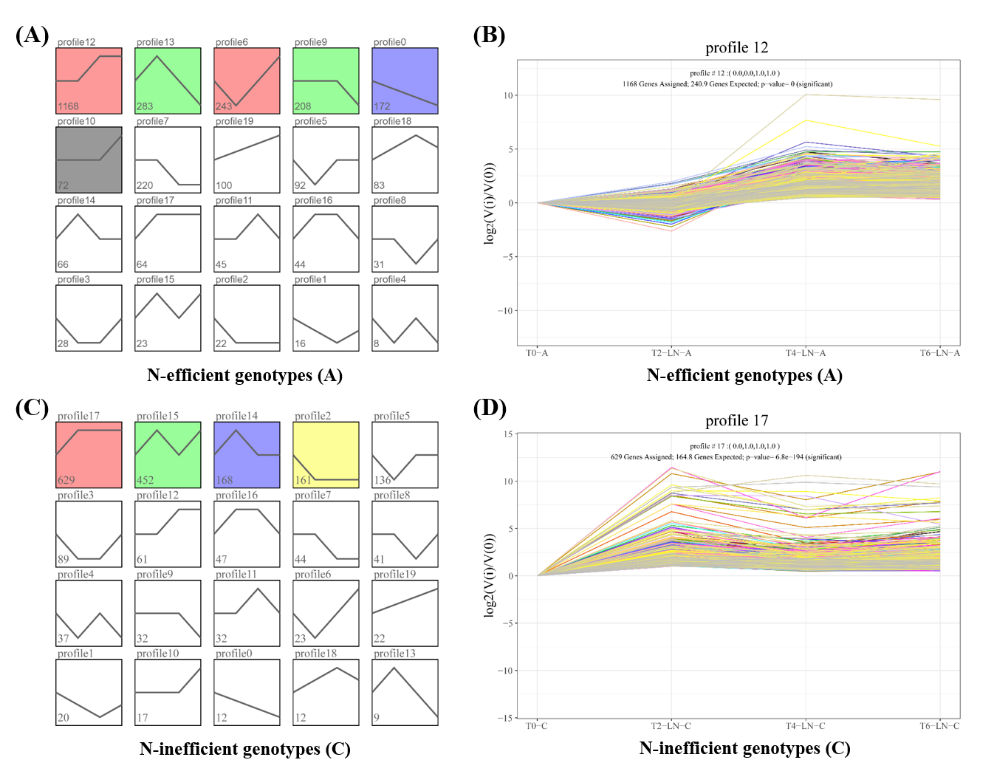
**Figure S9.** Gene expression patterns across four-time points (T0, T2, T4, and T6) in genotypes A and C under low N stress. (A) and (C) indicate the variation trend of differentially expressed genes (DEGs) in genotypes A and C, respectively. Above the box is the ID of the changing trend, and the number in the box indicates the number of DEGs contained in the trend. The grid with color indicates a significantly enrichment trend ( *p* < 0.05), and the closer the color is, the more similar the changing trend is. (B) and (D) represent the changing trend of genes in profile 12 with genotype A and profile 17 with genotype C, respectively.


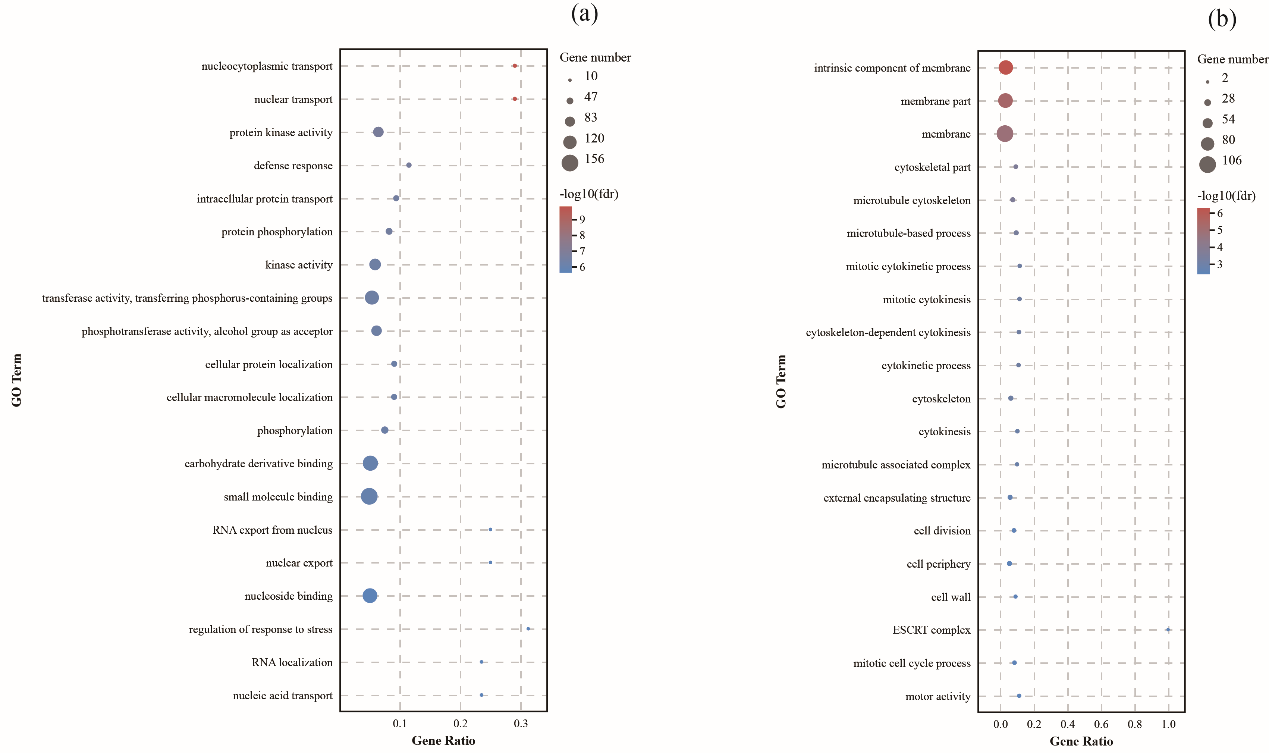


**Figure S10.** Top 20 gene ontology (GO) terms of differentially expressed genes (DEGs) in profile 12 of genotype A (a) and profile 17 of genotype C (b).


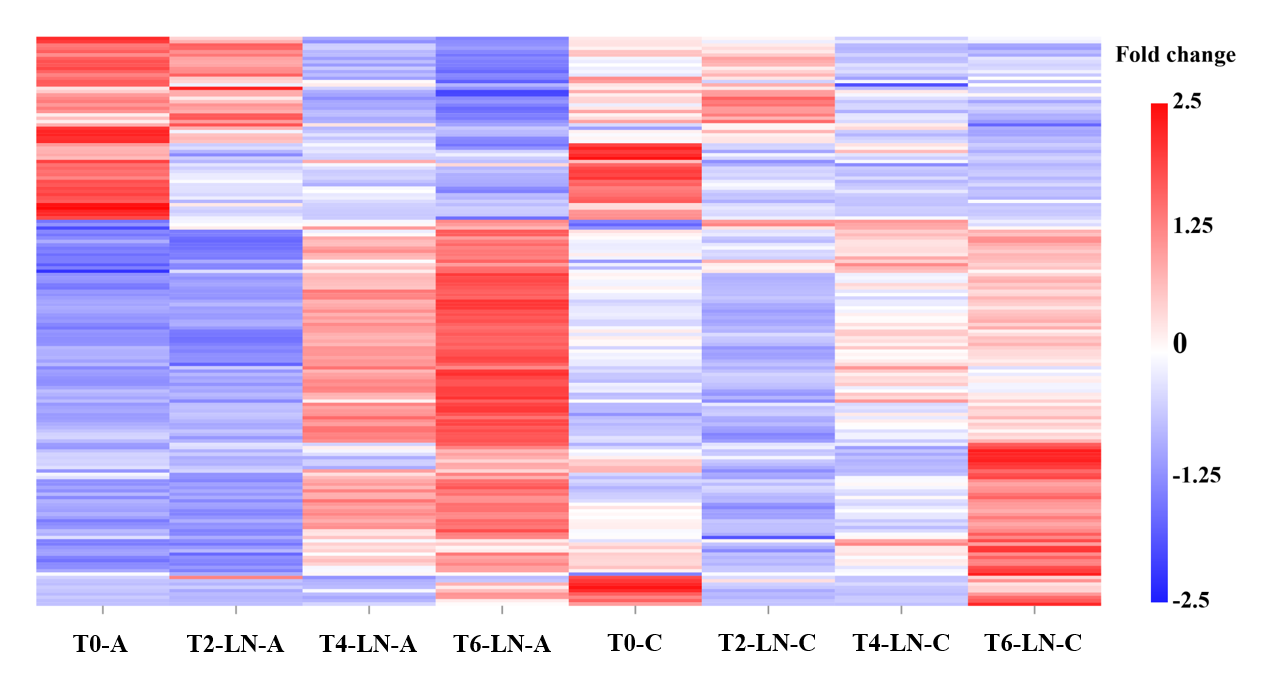


**Figure S11.** Expression pattern analysis of genes in the ‘magenta’ module. Red: upregulated; blue: downregulated. A: N‑efficient genotypes; C: N-inefficient genotypes. T0, T2, T4, and T6 represent 0, 5, 20, and 40 days of N treatment, respectively. LN: low N treatment.


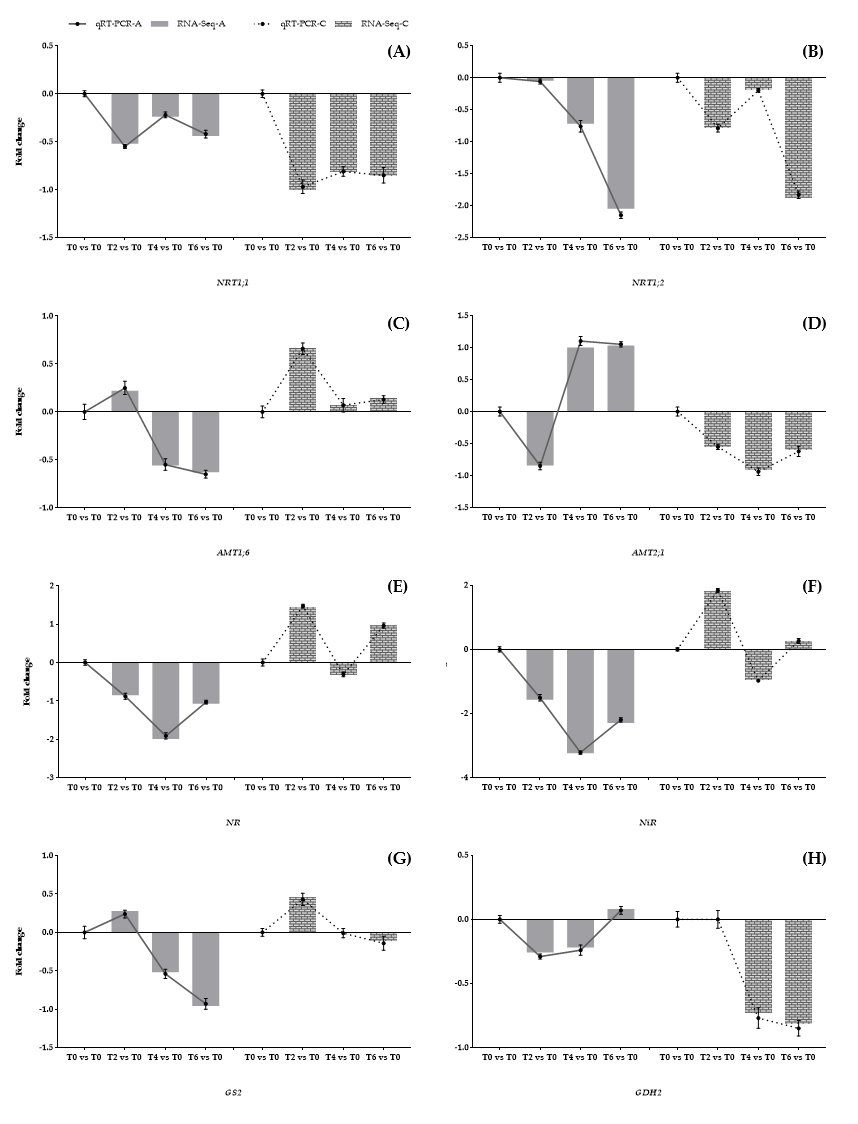


**Figure S12.** Expression of key genes in nitrogen metabolism in the leaves of genotypes A and C. (A-H) represent the expression trends of *NRT1;1*, *NRT1;2*, *AMT1;6*, *AMT2;1*, *NR*, *NiR*, *GS2,* and *GDH2*, respectively. The columns represent the results of RNA sequencing, and the lines show the qRT-PCR results. Vertical bars indicate SDs (n = 3) in the qRT-PCR analysis. A: N-efficient genotypes; C: N-inefficient genotypes.
